# Structure–Function Analysis of the Self-Sufficient CYP102 Family Provides New Insights into Their Biochemistry

**DOI:** 10.3390/ijms26052161

**Published:** 2025-02-28

**Authors:** Tiara Padayachee, David C. Lamb, David R. Nelson, Khajamohiddin Syed

**Affiliations:** 1 Department of Biochemistry and Microbiology, Faculty of Science, Agriculture and Engineering, University of Zululand, KwaDlangezwa 3886, South Africa; teez07padayachee@gmail.com; 2 Faculty of Medicine, Health and Life Sciences, Swansea University, Swansea SA2 8PP, UK; d.c.lamb@swansea.ac.uk; 3 Department of Microbiology, Immunology and Biochemistry, University of Tennessee Health Science Center, Memphis, TN 38163, USA; drnelson1@gmail.com

**Keywords:** cytochrome P450, CYP102, heme domain, redox partner, fatty acid, active site, mutant, hydrogen bond

## Abstract

Cytochromes P450 are a superfamily of heme-containing monooxygenases involved in a variety of oxidative metabolic reactions, primarily catalyzing the insertion of an oxygen atom into a C-H bond. CYP102 represents the first example of a bacterial P450 that can be classified as a type II (eukaryotic-like) P450 and functions as a catalytically self-sufficient enzyme. These unique features have made CYP102 an attractive system for studying P450 structure and function. However, an overall picture of the specific amino acid residues that are crucial to the functioning of CYP102 and the effect of mutations on the P450 structure and catalysis is yet to be reported. Such an approach will aid protein engineering approaches used to improve this enzyme. To address this research knowledge gap, we have investigated 105 CYP102 crystal structures in this study. We demonstrate that the CYP102 active site is highly dynamic and flexible. Amino acid residues that play critical roles in substrate binding, orientation, and anchoring were identified. Mutational studies highlighted the roles of amino acids and provided possible bioengineering improvement strategies for CYP102. Decoy molecules are a promising agent for deceiving CYP102 and permitting non-native substrates into the active site. Ru(II)-diimine photosensitizers and zinc/cobalt (III) sepulchrate (Co(III)Sep) could be used as alternative electron sources. The present study serves as a reference for understanding the structure–functional analysis of CYP102 family members precisely and of P450 enzymes in general. Significantly, this work contributes to the effort to develop an improved CYP102 enzyme, thereby advancing the field of P450 research and potentially leading to new industrial applications.

## 1. Introduction

Enzymes represent a versatile platform for catalyst development to solve challenging regio- and stereoselectivity problems in organic chemistry. Among the enzymes utilized in biotechnology, cytochrome P450 monooxygenases (CYPs/P450s) have been particularly researched over the past six decades due to their unique enzymatic properties and potential use in almost all areas of biology. P450s are a family of heme-containing proteins found across the domains of life and in viruses [1]. These enzymes are unique in biology as they perform catalytically diverse enzymatic reactions with chemo-, regio-, and stereoselectivity despite primarily being identified for their monooxygenase activity [2,3,4,5,6,7]. As well as monooxygenation reactions, P450s have been shown to catalyze epoxidation, heteroatom oxygenation and release, group migration, ring coupling and ring formation, ring contraction, dimer coupling, and aromatic dehalogenation [8]. Due to this unique enzymatic capability, P450s have been explored in all areas of biology ranging from drug discovery and drug metabolism to bioremediation, sensor development, bioethanol production, and human valuable chemical production [9,10,11,12,13,14].

To function, P450s require a source of electrons to perform their enzymatic reactions. Generally, these reducing equivalents are provided by ancillary redox partner proteins. At this time, P450 systems are classified into 10 different classes depending on the topology of the protein components involved in the electron transfer to the P450 enzyme [15]. The majority of bacterial and mitochondrial P450s belong to type I P450s, which contain a soluble P450 heme domain, an iron–sulfur protein, and a flavin adenine dinucleotide (FAD)-containing reductase [15]. Microsomal P450s are classified as type II P450s that comprise a membrane-anchored heme protein domain and anchored nicotinamide adenine dinucleotide phosphate (NADPH)-cytochrome P450 reductase domain, which contains both the FAD and flavin mononucleotide (FMN) binding domains in a single polypeptide chain [15].

CYP101 from *Pseudomonas putida* [16,17] and CYP102 (also known as P450 BM3) from *Bacillus megaterium* [18,19] are the most extensively studied P450s to date and are thus considered model P450s for understanding general P450 structure–function relationships. CYP102 is a 119 kDa protein and performs the hydroxylation of exogenous fatty acids, amides, and alcohols at the ω-1, ω-2, and ω-3 carbons with well-defined stereospecificity [19,20]. However, the endogenous role(s) of CYP102 remains unknown. CYP102 was found to be unique among bacterial P450s as it represents the first example of a bacterial P450 that can be classified as a type II P450 and is catalytically self-sufficient as the electron transfer FAD/FMN reductase domain and the P450 monooxygenase domain are contained in a single peptide [18,21]. Due to this nature, CYP102 is considered a model for microsomal P450s in higher organisms and this has been a primary focus since its discovery. CYP102 has the highest monooxygenation rate yet reported for a P450 enzyme [22]. It performs hydroxylation reactions with a high efficiency in the presence of NADPH and oxygen, and this can be explained by the fusion of the two domains, resulting in a high efficiency of intramolecular electron transfer [23].

Since the full-length CYP102 structure was still unknown, efforts to comprehend CYP102’s entire mechanism were put on hold. The CYP102 dimer’s interface was resolved by employing a number of techniques, including molecular modeling, chemical cross-linking, and negative stain 2D EM in conjunction with SEC–MALS analysis [24]. Four domains in various conformations were found by 2D analysis, demonstrating CYP102A1’s dynamic nature [24]. The structure of CYP102, a compact globular protein dimerized at its reductase domains and a dynamic heme domain exhibiting numerous conformations, is responsible for its high catalytic efficiency [24]. This dynamic structure was reaffirmed using a cryo-electron microscopy (cryo-EM) single-particle approach, where the full length of CYP102 was revealed and provided more insight into the electron delivery and mechanism of CYP102 catalysis [25]. The structure showed a homodimer where both the heme and FAD domains are in contact with each other [25]. The full-length structure existed in multiple conformations (open and closed) and was highly dynamic. In the closed conformation, the structure was densely packed and the heme and FAD domains remained in contact [25]. One FMN domain, however, interacts with the proximal side of the heme in the open conformation while the other FMN domain remains closed to its FAD [25]. For the effective transmission of electrons between the FMN domain and the heme domain, the two FMN domains alternate between open and closed conformations [25]. The FAD moiety is situated adjacent to the FMN domain in the closed conformation, and it can accept electrons and rotates and opens up toward the proximal side of the heme [25]. This movement can help create an active complex that delivers electrons to the heme. This indicates that the highly effective catalysis of CYP102A1 is significantly influenced by the protein dynamics of CYP102 heme domain.

Due to its high catalytic efficiency and its ability to act as a peroxygenase by substituting hydrogen peroxide for the reductase domain, cofactor, and oxygen [26], CYP102 has been extensively modified and adapted for various biotechnological applications. Different approaches, such as directed evolution, chimeragenesis, CASTing, and structure-led mutagenesis, have been used to engineer CYP102 capable of regio- and/or stereoselective synthesis of a variety of compounds including the production of oxidized derivatives of steroids, alkanes, and human drugs [27,28,29]. A combination of directed evolution and site-directed mutagenesis led to the generation of CYP102 variants capable of direct, regio- and enantioselective hydroxylation of linear alkanes [27]. Over the years, there was a surge in the development of CYP102A1 variants and, therefore, a downloadable database of over 1500 variants of CYP102A1 was developed [30]. The variants within this database are categorized by their catalyzed reactions and it includes information about the substrates which will assist other researchers to develop machine learning methods to accelerate reaction discovery [30]. A major challenge in synthetic organic chemistry is the oxidative C-H activation of complex organic compounds such as progesterone and testosterone [28]. A breakthrough has been achieved in this regard whereby CYP102 has been engineered by the CASTing method for regio- and stereoselective oxidative C-H activation and for the sustainable and green production of high-value hydroxy fatty acids (HFAs). [28,31]. CYP102 has been engineered to perform catalytic reactions identical to those performed by human P450 CYP2C19, e.g., a variant of CYP102 successfully performed a 5-hydroxylation reaction of omeprazole [32]. Further, a double variant of CYP102 showed oxidative activity toward diclofenac, ibuprofen, and tolbutamide [33]. This variant catalyzed the hydroxylation at specific positions, producing 4′-hydroxydiclofenac, 2-hydroxyibuprofen, and 4-hydroxytolbutamide [33]. The F87A variant of CYP102 showed enhanced peroxygenase activity whilst shifting hydroxylation further away from the terminal position [26]. Other CYP102 variants have also been used to resolve chemical synthetic issues involving enantioselective oxidation of simple terminal alkenes [34]. Two CYP102 variants were able to convert a range of terminal alkenes to either (R)– or (S)–epoxides with high catalytic turnovers and high epoxidation selectivity [34]. Another P450 variant revealed an enhanced and specific epoxidation activity for the terpenes geraniol and linalool [35]. CYP102 variants have the ability to produce vital intermediate compounds that are essential for the production of other metabolites, such as styrene oxide, which is vital in the production of antidiabetic agents and dopamine agonists [36].

A few amino acid CYP102 mutations unlocked significant cyclopropanation activity [37]. CYP102 has also been used in the biocatalytic preparation of structurally different steroidal C7β alcohols, and these compounds have shown significant promise as neuroprotective and anti-inflammatory agents in the treatment of chronic neuronal damage [38]. A CYP102 variant with seven mutations showed a high conversion rate of perillyl alcohol (POH) into its oxidative derivatives [39]. POH is a secondary metabolite of plants and is known for its anticancer properties. Structure-directed evolution of the CYP102 heme active site domain produced highly selective magnetic resonance imaging (MRI) probes for detecting neurological chemicals such as dopamine and serotonin [40]. Decoy molecules such as perfluorinated carboxylic acids (PFCs) are popular in the CYP102 research as they are known to “trick” the CYP102 enzyme and allow for the hydroxylation of non-natural P450 substrates [41]. Dual-functional small molecules (DFSMs) have also been used with CYP102 to explore product diversity of this engineered P450 [42]. The increasing popularity of this well-engineered P450 in many scientific fields led to the need to increase the speed of its crystallization. Therefore, the abietane diterpenoid derivative N-abietoyl-l-tryptophan (AbiATrp) was developed [43]. CYP102 is a highly evolvable catalyst and its variants have a wide range of molecular properties, including activity towards non-natural substrates and inorganic solvents.

The enormous significance of CYP102 in biology (as described above) has resulted in an explosion in the structure–function analysis of this P450, leading to more than 100 crystal structure depositions at the Research Collaboratory for Structural Bioinformatics Protein Data Bank (RCSB PDB) [44]. Structural information based on high-resolution CYP102 crystal structures complexed with various ligands revealed the role(s) of different amino acids in its structure–function analysis. However, a thorough and complete analysis of all the deposited crystal structures in order to understand specific amino acids’ role in CYP102 structure–function, particularly in the active site dynamics, still requires elucidation. Recently, such approaches have been undertaken with CYP107 and CYP121, whereby detailed analysis of crystal structures resulted in the identification of key amino acids critical to their structure–function [45,46]. Providing an overall picture of the specific amino acid residues crucial to the functioning of CYP102, the effect of mutations on the P450 structure and catalysis, and the different protein engineering approaches used to improve this enzyme will have great impacts in biocatalysis and biotechnology. In this paper, 105 crystal structures of CYP102 were analyzed, and the structure–function relationship of CYP102 is described, including the nature of this P450 in open and closed conformational states and a comprehensive analysis of amino acids’ interactions with various ligands and/or substrates. This work contributes to further efforts to develop an improved CYP102 enzyme that can be exploited to catalyze non-native substrates.

## 2. Results and Discussion

### 2.1. CYP102 Is Structurally Highly Dynamic

One hundred and thirty-six CYP102 crystal structures were retrieved from the RCSB PDB [44]. Among these crystal structures, a hundred and five are available for public use in the RCSB PDB (Table S3). Analysis of CYP102 crystal structures revealed that 54% of the resolved crystal structures are in the closed conformation (bound with ligand and heme), indicating this P450 has been crystallized with numerous ligands bound in the active site and the rest belonged to the open conformation (containing only heme cofactor) (Table S1).

The surface area for CYP102 open conformations ranged between 1004 Å^2^ and 2016 Å^2^, the volume ranged between 425 Å^3^ and 1611 Å^3^ and for the closed conformation the surface area ranged between 700 Å^2^ and 1961 Å^2^ and the volume ranged between 382 Å^3^ and 1700 Å^3^ (Table S1). Using the available crystal structure data, the average area and volume for CYP102 in an open conformation are 1510 Å^2^ and 1018 Å^3^ compared to 1331 Å^2^ and 1041 Å^3^ for a closed conformation. Therefore, the estimated change in surface area from the open to closed conformation is 179 Å^2^, reflecting the decrease in the area, and an increase in volume from open to closed conformation (23 Å^3^). The increase in volume may be due to the large bulky fatty acid substrates associated with this P450. A decrease in surface area is also observed in other P450s, such as CYP107 which oxidatively tailors large macrolide antibiotic precursors (Table 1) [45]. In contrast, an increase in volume has been observed in human drug-metabolizing P450s, such as CYP3A4 and CYP2A6, when bound to ketoconazole, erythromycin, and phenacetin, respectively [47,48]. Molecular dynamic analysis of amino acids in 105 CYP102 crystal structures revealed a high root-mean-square difference (RMSD) of 4.4 Å compared to CYP107FH5 (RMSD of 3.0 Å) and CYP121A1 (0.2 Å), providing supporting evidence for the idea of CYP102’s flexible nature (Table 1).

Analysis of CYP102 active site cavity amino acids revealed that residues that form the open conformation (42 amino acids) are found in the closed conformation, highlighting their importance in active site architecture maintenance (Table 2). However, three amino acids were unique to the closed conformation, namely, Trp72, Gln257, and Thr267 (Table 2). Additionally, 76% of the amino acids were conserved in all open conformations, whereas 69% were conserved in all closed conformations (Table 2). These results strongly point towards the dynamic nature of the amino acids within the active site of CYP102 during interactions with numerous ligands and, thus, the highest RMSD, as indicated in Table 1.

### 2.2. Analysis of the Heme, FMN, and FAD Domain Interactions Revealed the Possible Electron Transfer Pathway of CYP102

**Figure 1:**
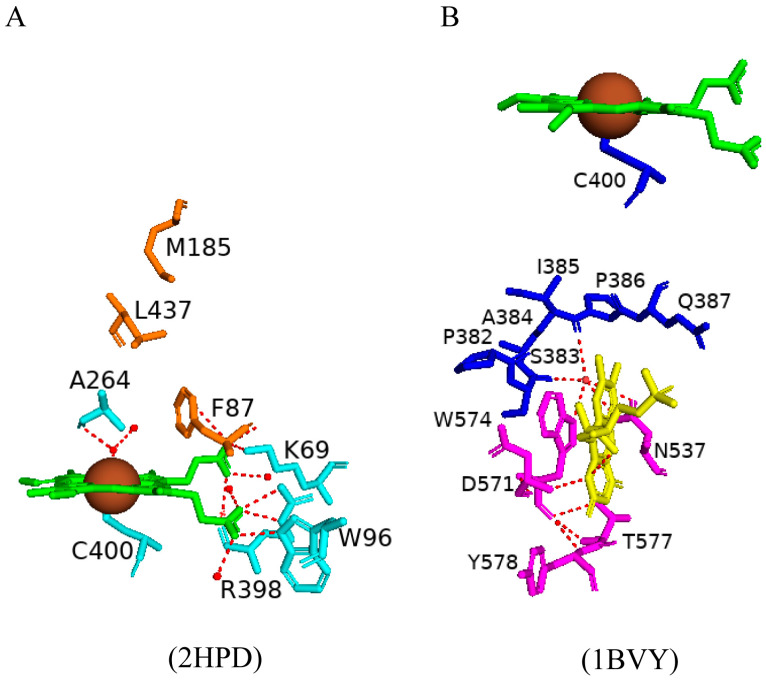
Analysis of CYP102 interactions within the heme (**A**) and FMN (**B**) domains, respectively. Heme is shown in green; FMN is shown in yellow; the iron molecule is shown as a brown sphere; the sixth water ligand is shown as a red dot. Amino acid residues sharing a direct polar interaction with the heme are shown in cyan, and amino acid residues sharing van der Waals interactions with the heme are shown in orange. The amino acid residues that are part of the heme domain are shown in blue, and the amino acid residues that are part of the FMN domain are shown in magenta. Polar interactions are indicated as red dashed lines; water molecules are represented as red dots, and amino acid residues are labeled according to their single-letter codes. PDB code is shown underneath the respective model.

CYP102 is a class II cytochrome P450 and interacts directly with a reductase partner containing FAD and FMN cofactors. The P450 heme is accessible via a long hydrophobic channel that is composed of mainly hydrophobic, non-aromatic amino acid residues [49]. The sixth water ligand forms hydrogen bonds with an additional water molecule and the carbonyl group of Ala264 (Figure 1A) [49]. The heme carboxylate group shares direct hydrogen bonds with Lys69, Trp96, and Arg398 (Figure 1A). There are also van der Waals interactions between the P450 heme and Phe87, Met185, and Leu437 [49] (Figure 1A). Phe87 may be important in substrate anchoring, whereas the latter two residues may be involved in stabilizing the active site. A closer look at the substrate-binding pocket revealed an adjacent hydrophobic patch that is exposed to the solvent containing residues Leu14, Leu17, and Ala91 [49]. These hydrophobic residues may be important for the initial interaction of a lipophilic substrate.

FAD cofactors serves as an electron acceptor from NADPH, whereas the FMN moiety interacts with and reduces the heme-bound P450 iron atom [50]. Analysis of the FMN and heme domains of CYP102 revealed interactions between the two domains. Trp574 of the FMN domain and Ser383 and Ile385 of the heme domain share a water-mediated hydrogen bond via one water molecule (Figure 1B). Trp574 is known to be critical for electron transfer from the FMN to the heme domain; therefore, electrons may flow from the FMN and/or Trp574 to the amino acids between Pro382 and Gln387 and then directly to the heme iron through bonded orbitals via Cys400 [50]. The CYP102-FMN complex may be strengthened by long-range electrostatic interactions between charged surfaces on both molecules; this is evident by the orientation of the FMN domain to the heme domain as the negatively charged amino acids of the FMN domain are assembled on the side that faces and interacts with the positively charged proximal side of the heme domain [50].

The structure of the FAD domain of CYP102 is important in understanding its interactions with its associated FMN domain and the nature of the binding sites. Three conserved residues, often referred to as a “catalytic triad” of residues (Ser831, Cys999, and Asp1044) are found in the FAD domain and are known to play an important role in the binding of NADPH and the regulation of electron transfer from NADPH to the FAD moiety [51].

**Figure 2:**
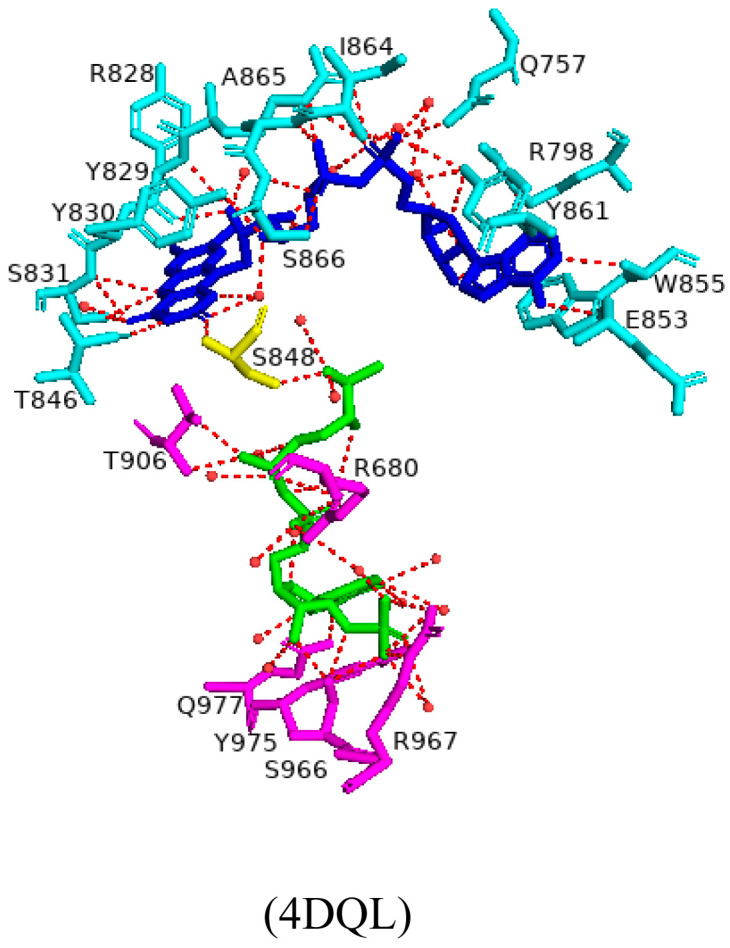
Analysis of interactions between the FAD domain and NADPH-binding domains of CYP102. FAD moiety is shown in blue, NADPH in green, and connecting residue in yellow. Amino acid residues sharing a direct polar interaction with the FAD domain are shown in cyan, and amino acid residues sharing polar interactions with the NADPH moiety are shown in magenta. Polar interactions are indicated as red dashed lines; water molecules are represented as red dots, and amino acid residues are labeled according to their single-letter codes. PDB code is shown underneath the respective model.

The FAD moiety shares direct hydrogen bonds with multiple amino acid residues and nine water molecules (Figure 2). Key interactions include hydrogen bonding of the FAD adenine base to Glu853 and Trp855 (Figure 2). The adenine moiety also shares multiple van der Waals interactions with residues Arg797, Val849, and Ala853 [51]. The pyrophosphate oxygen shares hydrogen bonds with seven amino acid residues (Gln757, Arg828 and Tyr861, Tyr828, Tyr829, Ile863, and Ala864) (Figure 2) [51]. Adjacent tyrosine residues (Tyr829 and Tyr830) form hydrogen bonds with the FAD ribityl hydroxyl groups, and the FAD isoalloxazine ring interacts with Ser831, Thr846, and Ser848 via hydrogen bonds (Figure 2) [51]. Analysis of the FAD–NADPH bound complex revealed various important protein interactions with the NADPH 2′-phosphate that aid in the strong selectivity for NADPH instead of NADH [51]. The structure of the NADPH-binding site further supports this selectivity, with polar interactions between the adenine ribosyl 2′-phosphate and Ser966, Arg967, Tyr975, and thirteen water molecules (Figure 2). These interactions are highly conserved in other ferredoxin–NADP+ reductase (FNR) and diflavin reductase family members [51]. The connecting residue between the FAD domain and the NADPH moiety is Ser848 which shares a hydrogen bond with both domains and may be involved in the electron transfer pathway of CYP102.

### 2.3. CYP102 Interaction with Numerous Ligands Reveals Amino Acid Residues Playing a Role in Efficient Substrate Binding

#### 2.3.1. Tyr51 May Play a Role in Substrate Anchoring of Palmitoleic Acid and N-Palmitoylglycine

**Figure 3:**
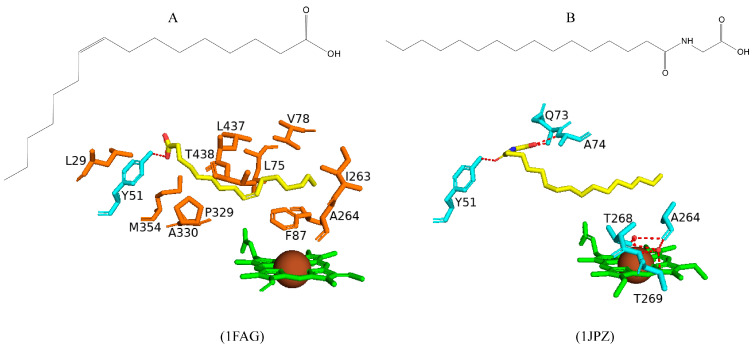
Analysis of CYP102 interactions with palmitoleic acid (**A**) and N-palmitoglycine (**B**). Substrates are colored with carbon atoms as yellow, nitrogen as blue, and oxygen as red; heme is shown in green; iron molecule is shown in brown. Amino acid residues sharing a direct polar interaction with the substrates are shown in cyan, and amino acid residues sharing hydrophobic interactions with the substrates are shown in orange. Polar interactions are indicated as red dashed lines; water molecules are represented as red dots, and amino acid residues are labeled according to their single-letter codes. The chemical structure of the substrate is shown above the protein model, and the PDB code is shown underneath the respective model. Amino acid residues within 5 Å of the ligand are shown in Table S2.

CYP102 can perform an epoxidation reaction at the C9-C10 double bond in palmitoleic acid; this double bond is situated far away from the heme iron; therefore, rearrangement of the substrate is required to bring the double bond closer to the heme iron for the reaction to occur [52]. The crystal structure of palmitoleic-acid-bound CYP102 provided information on the critical interactions between the carboxylate group of palmitoleic acid and Tyr51 (Figure 3A). If the carboxylate moiety of the substrate is deprotonated and unrestricted it moves further into the active site and allows for the epoxidation reaction to occur [52]. It is interesting to note that palmitoleic acid is bound in the distal pocket of the active site and far away from the heme iron (Figure 3A). The alkyl end of the substrate curls away from the heme due to the Phe87 residue that is known to block the substrate from coming close to the heme iron by adopting an almost parallel conformation to the heme iron, effectively blocking the methyl group from approaching and it is thus trapped by various hydrophobic residues [52] (Figure 3A). This explains why CYP102 never hydroxylates this end of fatty acid substrates. The structure also revealed a closure of the active site channel, which highlights the CYP102 conformational changes induced by substrate binding [52].

Long-chain N-acyl amino acids are considered to act as physiologically active signalling molecules. N-palmitoylglycine has been shown to modulate monoamine levels in the hippocampus [53]. Due to its increased solubility in aqueous solution, N-palmitoylglycine reduces many experimental-related issues that prevent studies on high concentrations of a substrate in P450 crystallography studies [53]. Hence, these properties make N-palmitoylglycine an ideal tool for investigating the CYP102/substrate complex. This crystal structure was crystallized in a higher resolution compared to the substrate complex with palmitoleic acid and revealed critical information regarding the structural changes that occur upon substrate binding [53]. The majority of these changes occur in the ‘lid’ domain of CYP102; this domain consists of the F and G helices and the loop between them. Upon substrate binding, this domain slides towards the loop between the F and G helices, and due to direct contact between the B’ helix and the G helix, the B’ helix moves with the lid domain [53]. Overall, it seems that these dynamic motions position the substrate in the active site for the reaction’s subsequent stages. N-palmitoylglycine binds to CYP102 with a largely increased affinity (Kd of 262 nM) compared to palmitic acid (Kd of 2 µM). Therefore, N-palmitoylglycine can be considered as a substrate for CYP102 [53].

N-palmitoylglycine is oriented in the CYP102 active site in a similar fashion to palmitoleic acid (Figure 3B). N-palmitoylglcyine does not contain a double bond between C9–C10 and, therefore, extends slightly more into the active site of CYP102 (Figure 3B). Both substrates share a direct hydrogen bond with Tyr51, and thus, the alkyl moieties are in similar positions (Figure 3B). The glycine carboxylate group forms two direct hydrogen bonds with Gln73 and Ala74 (Figure 3B), bringing the negatively charged portion of the substrate closer to the B’ helix which incurs more favorable electrostatic interactions resulting in increased binding affinity [53]. The CYP102 I helix has a kink due to a bound water molecule. This water molecule is displaced upon substrate binding and results in further hydrogen bonds forming between adjacent residues in the I helix and causing it to become more linear in structure [53]. This rearrangement results in the displacement of the sixth water ligand and, a hydrogen bonding network with the hydroxyl group of Thr268, the carboxyl group of Ala264, two water molecules, and a water-mediated hydrogen bond with Thr269 form (Figure 3B). The sixth water displacement from the iron atom permits an oxygen molecule to bind following reduction [53]. Despite displacement, the sixth water ligand remains close to the heme iron and may be involved in the catalytic reaction steps that follow.

#### 2.3.2. Steric Hindrance Between Phe87, Ala264, and N-(ω-Imidazolyl fatty acyl)-L-leucine Reveals Interactions That May Improve the Binding Affinity Between Inhibitors and CYP102

Imidazole inhibits many P450s because its nitrogen-containing ring is an excellent ligand to bind the heme iron [54]. Bound imidazole prevents P450s from binding oxygen, which is necessary for activity. However, imidazole is not a good CYP102 inhibitor due to steric conflicts within the active site [55]. Although other inhibitors have been generated to increase the enzyme’s affinity by including an imidazole moiety in a fatty acyl chain, the affinity for CYP102 is weak [55]. To further test the CYP102 flexibility, active site N-(ω-imidazolyl fatty acyl)-L-amino acid inhibitors were developed. Addition of an L-leucine head group to the inhibitor led to an increased binding affinity (5.2 μM) [55]. This derivative was then introduced into a mixture of CYP102 and palmitic acid which interestingly led to a complete conversion of the heme iron from low to high spin. This result indicated that the derivative bound to CYP102 despite the presence of palmitic acid and exhibited higher affinity compared to the native substrate [55].

**Figure 4:**
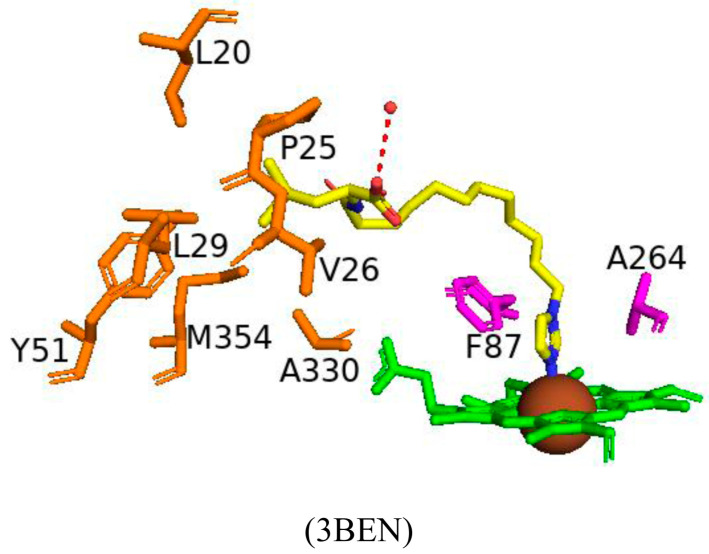
Analysis of CYP102 interactions with an inhibitor, N-(12-Imidazolyldodecanoyl)-L-leucine. The inhibitor is colored with carbon atoms as yellow, nitrogen as blue, and oxygen as red, the heme is shown in green, and the iron molecule is shown in brown. Amino acid residues sharing hydrophobic interactions with the inhibitor are shown in orange. Amino acids having a steric conflict with the inhibitor are shown in magenta. Polar interactions are indicated as red dashed lines; water molecules are represented as red dots, and amino acid residues are labeled according to their single-letter codes. PDB code is shown underneath the respective model. Amino acid residues within 5 Å of the inhibitor are shown in Table S2.

The inhibitor is very similar in structure to N-acyl amino acids. However, the structure of CYP102 still resembles the open conformation despite the bound inhibitor [55]. As previously mentioned, the carboxylate group of N-palmitoylglycine is close to the CYP102 B’ helix and results in favored interactions. However, the carboxylate group of the derivative faces away from the B’ helix. The leucine moiety is situated in a hydrophobic pocket containing seven amino acid residues, Leu20, Pro25, Val26, Leu29, Tyr51, Ala330, and Met354, and shares a direct hydrogen bond with one water molecule [55] (Figure 4). The imidazole ring of the inhibitor derivative is almost parallel to the I helix. Phe87 is highly conserved within CYP102s and appears to greatly limit spatially the active site in close proximity to the heme [55]. The orientation of the imidazole ring causes a steric conflict with Phe87 and Ala264 and results in a perturbed ability to occupy the sixth water ligand position [55] (Figure 4). These results have uncovered several approaches to design more potent inhibitors of CYP102. Adding a hydrophobic surface to the inhibitor may be beneficial by adding a longer acyl chain, resulting in more favored interactions, thus increasing binding affinity [55]. The addition of a longer acyl chain may permit the amino acid carboxylate group to interact with the B’ helix. These results reveal new interactions and suggest ways to improve the inhibitor binding affinity.

#### 2.3.3. Phe87 Increases the Solvent Tolerance and Protects the Heme of CYP102

**Figure 5:**
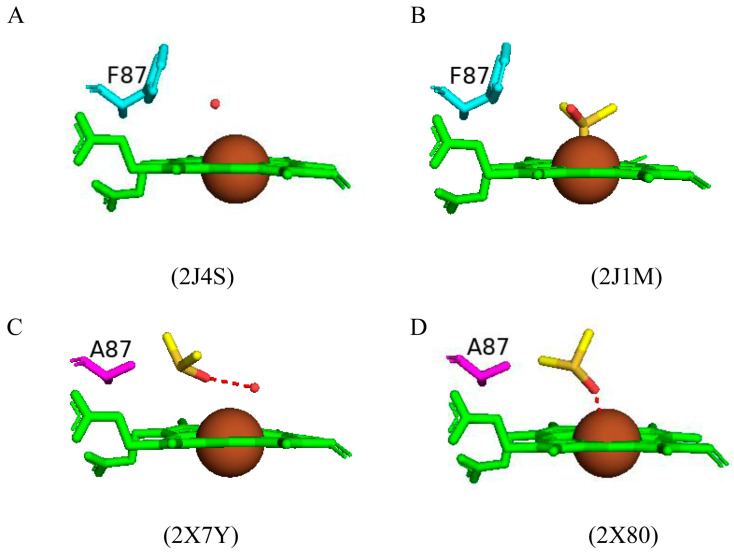
Analysis of CYP102 interactions with dimethylsulfoxide (DMSO). Wild type and 14% (*v*/*v*) DMSO (**A**), Wild type and 28% (*v*/*v*) DMSO (**B**), F87A variant and 14% (*v*/*v*) DMSO (**C**), and F87A variant and 28% (*v*/*v*) DMSO (**D**). Ligand is colored with carbon atoms as yellow, sulfur as light brown, and oxygen as red; heme is shown in green; the iron molecule is shown in brown. Wild-type amino acid residues are shown in cyan. Mutated amino acids are shown in magenta. Polar interactions are indicated as red dashed lines; water molecules are represented as red dots, and amino acid residues are labeled according to their single-letter codes. PDB code is shown underneath the respective model. Amino acid residues within 5 Å of the ligand are shown in Table S2.

Utilizing CYP102 as a biocatalyst for industrially important chemical reactions such as the hydroxylation of alkanes and/or alcohols requires an improved tolerance towards organic co-solvents and more research is needed on the effects of this co-solvent on the CYP102 enzymatic activity [56]. Dimethylsulfoxide (DMSO) is a polar organic co-solvent that can be added to boost substrate solubility and increase catalytic efficiency. CYP102 was bound with DMSO at different concentrations (14% (*v*/*v*) and 28% (*v*/*v*)) [56]. CYP102 retains 70% activity at the lower concentration and it drastically drops to 10% at the highest concentration [56]. This drastic drop is likely due to DMSO perturbing the heme coordination and resulting in a loss of activity. In the lower DMSO concentration, the sixth water ligand is displaced and no DMSO molecule was found bound within the active site (Figure 5A) whereas in the higher concentration of DMSO, a DMSO molecule is directly coordinated to the heme iron via a sulfur atom and is partially stabilized by hydrophobic interactions with Phe87 (Figure 5B). The I helix in the DMSO higher concentration is slightly bent and may provide additional room for the DMSO molecule to bind [56]. These results further highlight CYP102 molecular flexibility and the dynamic nature of the amino acid residues within the CYP102 active site.

To further understand the mechanism of DMSO inactivation and the pivotal role of Phe87 in CYP102 function, the phenylalanine residue was replaced with alanine by site-directed mutagenesis to develop an F87A variant [57]. This variant was crystallized with the same concentrations of DMSO. A DMSO molecule was bound within the active site at both DMSO concentrations (Figure 5C,D). At the lower DMSO concentration, the DMSO molecule was oriented with its oxygen atom towards the heme iron and shared a direct hydrogen bond with the sixth water ligand (Figure 5C). At the higher concentration, a DMSO molecule is directly coordinated with the heme iron, and no water molecule was found in close proximity to the heme iron (Figure 5D). This may be the reason CYP102 is inactivated at high levels of DMSO. The F87A variant’s catalytic activity was 33% in the presence of 14% (*v*/*v*) DMSO and only 2% enzymatic activity at a 28% (*v*/*v*) DMSO concentration [57]. These findings indicated that the variant had a lower tolerance to DMSO than the wild type. The comparative analysis between the wild type and variant suggests that the bulky phenyl side chain of Phe87 protects the heme moiety by modulating the accessibility of DMSO to the heme iron atom [57].

### 2.4. Identifying Amino Acids Involved in the Catalytic Function of CYP102

#### 2.4.1. Phe393 Thermodynamically Controls the Heme Environment

**Figure 6:**
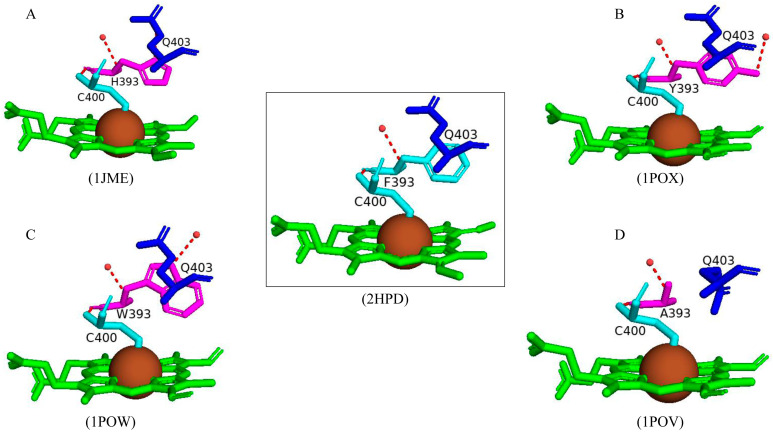
Analysis of Phe393’s role in CYP102 catalytic activity. Phe393 variants of CYP102 include F393H (**A**), F393Y (**B**), F393W (**C**), and F393A (**D**). Heme is shown in green; the iron molecule is shown in brown. Wild-type amino acid residues are shown in cyan and blue. Mutated amino acids are shown in magenta. A wild-type enzyme is shown in the box. Polar interactions are indicated as red dashed lines; water molecules are represented as red dots, and amino acid residues are labeled according to their single-letter codes. PDB code is shown underneath the respective model.

Phe393 is highly conserved in most P450s, including CYP102 [58,59]. However, some P450s, including CYP8 [60] and CYP74 [61], do not contain this highly conserved residue and interestingly function without activating molecular oxygen. This suggests that this residue plays a role in controlling the reaction between the heme iron and molecular oxygen [58,59]. Substitution of this residue was carried out to analyze its role in the P450 catalytic cycle. The CYP102 F393H variant revealed no major changes to the enzyme substrate specificity and the regiospecificity of the fatty acid hydroxylation reaction [58,59]. Despite no significant differences, the F393H variant was catalytically inefficient compared to the wild type. There were no significant structural differences besides the substitutions between the variants and the wild type except for the F393A variant (Figure 6). The conformation of Gln403 was different compared to all variants and the wild type; the amide moiety of Gln403 usually points away from the heme; however, in the F393A variant, the amide moiety occupies the space left by the bulky side chain of the phenylalanine residue (Figure 6D). Spectroscopic analysis reveals the effect the mutations have on the thermodynamic control of the heme iron. This control is a combination of many factors, including the location of Phe393 being extremely close to the heme iron and the ligated cysteine residue as well as sharing favorable interactions and the ability to protect the heme from exposure to the solvent [58,59].

#### 2.4.2. Ala264 Controls the Conformational Equilibrium of CYP102

**Figure 7:**
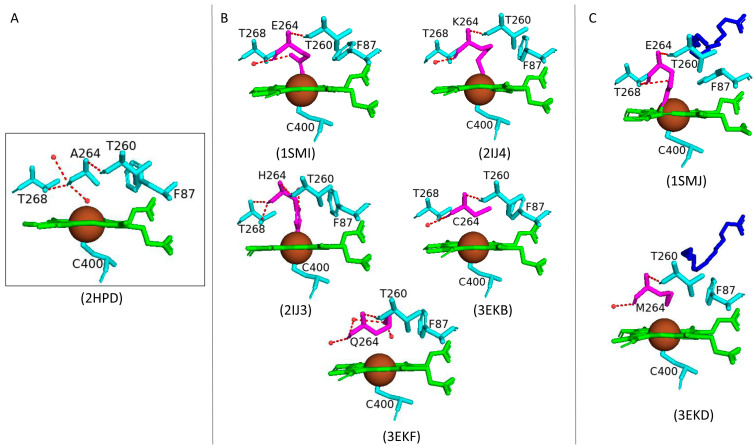
Analysis of Ala264’s role in CYP102 catalytic activity. CYP102 structures include a wild type (**A**) and variants such as A264E, A264K, A264H, A264C, A264Q (**B**), A264E bound with palmitoleate, and A264M bound with palmitate (**C**). Heme is shown in green; iron molecule is shown in brown; ligand is shown in blue. Wild-type amino acid residues are shown in cyan, and mutated amino acids are shown in magenta. Polar interactions are indicated as red dashed lines; water molecules are represented as red dots, and amino acid residues are labeled according to their single-letter codes. PDB code is shown underneath the respective model. Amino acid residues within 5 Å of the ligand are shown in Table S2.

When using P450s to produce oxy-functionalized organic molecules on a large scale, it is important to take into account the possibility of P450 inactivation by the complete removal of the heme macrocycle from the protein matrix or by a loss of heme iron ligation [62]. Upon fatty acid binding, CYP102 undergoes major conformational changes, mainly involving the reorganization of the P450 I helix and displacement of the sixth water ligand. The wild-type open conformation has a direct hydrogen bond between the sixth water ligand and Ala264 (Figure 7A).

Many substitutions of the Ala264 residue were made to further understand its role in the displacement of the sixth water ligand and conformational changes associated with substrate binding (Figure 7B). The A264E variant revealed CYP102 to be in a closed conformation despite the absence of a substrate [63]. This finding was due to residues occupying a conformation similar to the wild-type closed conformation (Phe87) and the displacement of the sixth water ligand (Figure 7B). Incorporating the mutation Glu264 in the wild-type structure leads to steric conflicts with many residues, and therefore, the mutation results in the destabilization of the open conformation; hence, the closed conformation is more suitable [63]. Despite the closed conformation associated with the A264E variant, it revealed a low spin state of the heme iron, indicating that a spin-state shift is a direct result of substrate binding [63].

Comparative analysis between the A264E variant with the A264K and A264H variants revealed the importance of this amino acid side chain and how it influences the conformation of CYP102. A264H variant resembled the open conformation of the wild-type enzyme. In contrast, the A264K variant resembled the closed conformation of CYP102 [64]. The presence of Lys264 led to direct iron coordination, and the I helix was pushed up in a similar manner to that observed in the A264E variant, leading to a conformational shift (Figure 7B). The structures of glutamic acid and lysine are generally comparable and thus lead to similar orientations when coordinated to the heme iron, sharing a direct hydrogen bond with the displaced sixth water ligand (Figure 7B). Interestingly, despite the heme coordination with His264, this variant adopts the open conformation, and the sixth water ligand is not displaced. This may occur due to the steric conflicts of the imidazole ring of histidine, and thus, the open conformation is preferred [64] (Figure 7B). However, despite the adopted conformations of both variants (A264K and A264H), they were deemed inactive as fatty acid substrates did not induce a spectral shift in the iron state [64]. Therefore, these enzymes were in the hexa-coordinated state, and neither oxygen nor a fatty acid substrate could displace the nitrogenous ligands, unlike the A264E variant where NADPH-dependent fatty acid hydroxylation did occur at reduced levels compared to the wild type [64].

The A264C and A264Q variants were strikingly different as this mutated amino acid residue did not coordinate with the heme iron (Figure 7B). However, the sixth water ligand was displaced in both variants, in the closed conformation without a substrate (Figure 7B). A264C and A264Q variants revealed a low spin state of the heme iron and once again provided evidence that substrate binding initiates the spectral shift of the heme iron [63]. The A264E variant bound with palmitoleate revealed Glu264 to be directly coordinated to the heme iron accompanied by the complete displacement of the sixth water ligand. Additionally, the Phe87 residue was tilted to prevent steric clashes with the bound substrate [62] (Figure 7C). The crystal structure of the A264M variant bound with palmitate revealed the complete displacement of the sixth water ligand and the orientation of the Phe87 residue was perturbed to accommodate the substrate (Figure 7C). These results reveal the critical role that Ala264 plays in terms of the conformational equilibrium of CYP102 between the open and closed conformation as well as novel heme iron ligand sets between the amino acid residue at the 264th position and the heme iron which led to the displacement of the sixth water ligand [62]. Therefore, it can be concluded that the CYP102 conformational change is not merely due to the displacement of the sixth water ligand but also the interaction with Ala264 [63].

#### 2.4.3. Thr268 Is Critical for the Overall Efficient Functioning of CYP102

**Figure 8:**
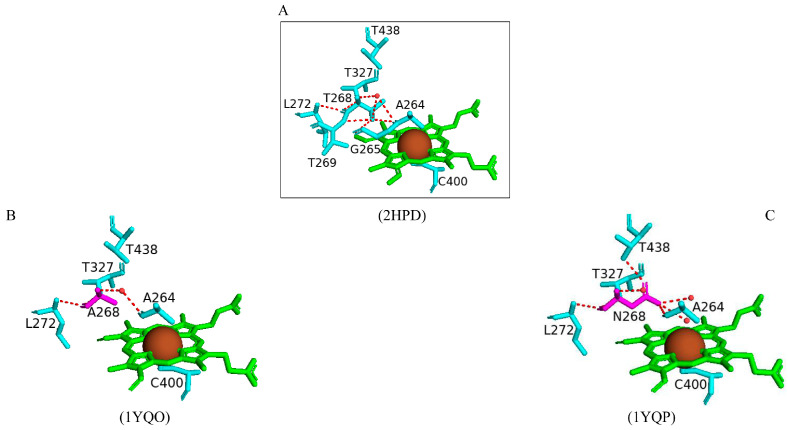
Analysis of Thr268’s role in CYP102 catalytic activity. CYP102 structures include the wild type (**A**) and variants such as T268A (**B**) and T268N (**C**). Heme is shown in green; the iron molecule is shown in brown. Wild-type amino acid residues are shown in cyan, and mutated amino acids are shown in magenta. Polar interactions are indicated as red dashed lines; water molecules are represented as red dots, and amino acid residues are labeled according to their single-letter codes. PDB code is shown underneath the respective model.

Thr268 adopts a different role within each P450 family depending on the nature of the substrate and the flexibility of the substrate-binding pocket [65]. These roles include proton donation, oxygen activation, and substrate recognition [65]. To understand the role of Thr268 in CYP102, mutations of this residue (T268A and T268N variants) were designed and the effects on the overall function of CYP102 were probed [65]. The alanine substitution removed the hydroxyl group and thus removed its ability to form hydrogen bonds and donate protons. In contrast, the asparagine substitution retains the possibility to form hydrogen bonds but eliminates the ability of this residue to donate protons [65]. In the wild-type enzyme, Thr268 forms many hydrogen bonds with adjacent amino acid residues, including Ala264, which is known to interact with the sixth water ligand (Figure 8A). In the T268A variant, the only significant difference was the mutation itself; it retained the hydrogen bond with Leu272 and a water molecule, which is hydrogen bonded to the carbonyl oxygen atom of Ala264 (Figure 8B). In the T268N variant, the Asn268 hydroxyl group shares a direct hydrogen bond with the sixth water ligand, Ala264, and an additional water molecule (Figure 8C). The oxygen moiety of the side chain shares a direct hydrogen bond with the hydroxyl group of Thr438. This residue adopts a different conformation than the T268A variant and wild-type enzyme (Figure 8C) [65]. The change in conformation is due to the prevention of steric hindrances between Asn268 and the methyl group of Thr438 (Figure 8C).

Spectral studies revealed that generation of the T268A variant led to lower concentrations of high-spin protein but almost negligible amounts for the T268N variant [65]. These substitutions led to a drastic decrease in the ability to generate the high-spin state of the enzyme upon substrate binding and an overall reduction in the shift activation and substrate recognition [65]. Both variants generated enzymes which have significantly lower turnover rate constants, lower degrees of productive coupling, and less stable oxy-ferrous species [65]. The CYP102 I helix undergoes many changes during the catalytic cycle of this P450. Thr268 is found within this helix and thus may act as a hydrogen bond acceptor and stabilize the hydroperoxy species. Alanine prevents this from occurring due to the absence of a hydroxyl group, and the hydrogen bond between the sixth water ligand and Ala264 is therefore weaker; hence, the sixth water ligand bound to the heme iron may be more favorable [65]. Interestingly, despite asparagine having the ability to donate and form a hydrogen bond, the NH_2_ group is facing the active site of CYP102 and forms a hydrogen bond with Ala26. Consequently, the mutant protein will not have the ability to accept a hydrogen bond from the hydroperoxy species and this may explain the uncoupling nature of this variant (Figure 8C) [65]. These results suggest that substituting the threonine residue leads to a highly uncoupled P450 catalytic cycle and therefore an inefficiently functioning enzyme.

#### 2.4.4. Ile401 and Ala330 Substitutions to Proline Enhanced CYP102 Activity

**Figure 9:**
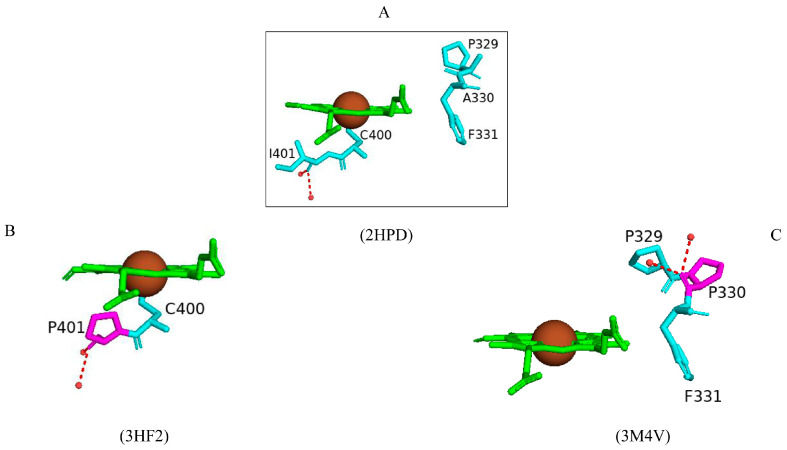
Analysis of active site cavity interactions of I401P and A330P of CYP102. CYP102 structures include the wild type (**A**) and the variant enzymes such as I401P (**B**) and A330P (**C**). Heme is shown in green; the iron molecule is shown in brown. Wild-type amino acid residues are shown in cyan, and mutated amino acids are shown in magenta. Polar interactions are indicated as red dashed lines; water molecules are represented as red dots, and amino acid residues are labeled according to their single-letter codes. PDB code is shown underneath the respective model.

Despite the nature of proline being a structurally disruptive residue which may lead to protein misfolding, some of the CYP102 proline variants had enhanced activity [66]. One of the success stories was the mutation of Ile401 to Pro401 [66]. In the wild-type enzyme, Ile401 is located on the C-terminal side of the cysteine-ligated residue (Figure 9A). The crystal structure of this variant revealed that the hydrogen bond network with water molecules is analogous in the wild type and in the variant enzymes (Figure 9B). When bound to lauric acid, the mutant enzyme displayed a 50% high-spin state and resembled characteristics of substrate-bound spectra [66]. The coupling and regioselectivity of this variant did not significantly change. However, the amount of peroxide formed decreased [66]. Non-natural substrates were probed for enzyme binding and activity analysis with variants, including (+)-a-pinene, fluorene, 3-methylpentane, and propylbenzene. The NADPH consumption rate, coupling, and product formation rate significantly increased for propylbenzene-bound enzyme compared to the wild-type enzyme [66]. The I401P variant also had higher product formation rates for fluorene, 3-methylpentane, and (+)-a-pinene. A point to be noted is that the wild-type enzyme does not metabolize (+)-a-pinene [66]. The I401P variant showed great potential to act as a powerful rate accelerator for non-natural substrates of CYP102.

In striking contrast, the A330P variant enzyme was 100% in the low-spin state and only showed increased catalytic activity towards small substrates such as pentane and toluene [67]. This mutation caused a perturbed orientation of Pro329 whereby the side chain was displaced and occupied the substrate access channel (Figure 9C). This probably restricts the active site of this variant, hence the resultant low-spin state [67]. The Phe331 residue remains in the same position as in the wild-type enzyme (Figure 9C). These results indicate that proline substitutions may produce variant enzymes with activity-enhancing properties, but this solely depends on the correct position of the specific substitution.

#### 2.4.5. Leu86, Ile401, and Phe261 Mutations to Glutamate Reveal Their Effects on Thermodynamic and Substrate Binding in CYP102

The majority of cytochrome P450s contain b-type heme cofactors that are covalently bound to the protein via linkages with the axial ligands to the heme iron [68]. Studies have revealed that, in CYP4 enzymes, the heme methyl group is covalently linked to a conserved glutamate residue [69]. Therefore, this raised the question of whether incorporating a glutamate residue close to the heme methyl group will also stabilize heme in other P450s. Three CYP102 amino acid residues close to the heme group that do not have major functional roles were substituted with glutamate (L86E, I401E, and F261E) to explore the ability of the carboxylate moiety of the glutamate residue to form an ester linkage with the heme methyl group [70].

**Figure 10:**
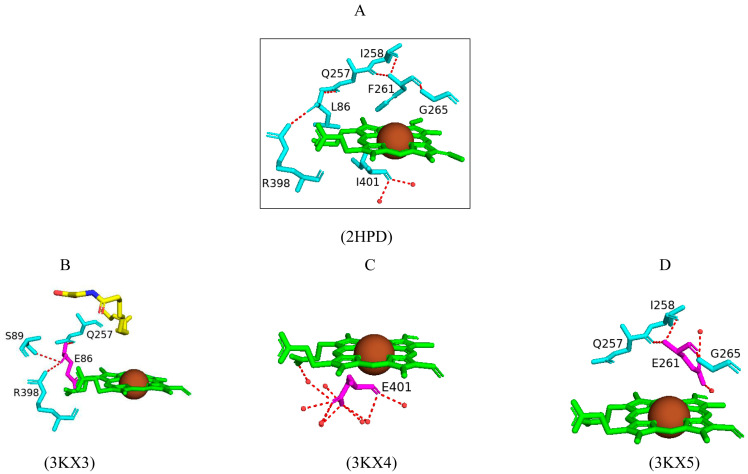
Active site cavity analysis of glutamate-substituted variants of CYP102. CYP102 structures include a wild type (**A**) and the variant enzymes such as L86E (**B**), I401E (**C**), and F261E (**D**). Heme is shown in green; the iron molecule is shown in brown; the substrate is colored with carbon atoms as yellow, nitrogen as blue, and oxygen as red. Wild-type amino acid residues that share a polar interaction are shown in cyan, and mutated amino acids are shown in magenta. Polar interactions are indicated as red dashed lines; water molecules are represented as red dots, and amino acid residues are labeled according to their single-letter codes. PDB code is shown underneath the respective model. Amino acid residues within 5 Å of the ligand are shown in Table S2.

The F261E and I401E variants displayed a low-spin state of the heme iron, whereas the L86E revealed a small proportion of the protein to be in the high-spin state. These results correlated with the resolved crystal structures as F261E and I401E variants were crystallized only in the open conformation in contrast to L86E which was only crystallized with bound substrate, N-palmitoglycine (Figure 10) [70]. The L86E variant was structurally similar to the wild-type enzyme bound with N-palmitoglycine (Figure 3B). There were no significant structural changes induced by the introduction of the glutamate residue besides the substitution itself (Figure 10A) [70] and the hydrogen bond between Gln257 and Arg398 in the wild-type enzyme is also observed in the variant enzymes (Figure 10A,B). Glu86 shares a direct hydrogen bond with Ser89 and the heme propionate group, but no interactions were observed between the residue and the heme methyl group (Figure 10B). The interactions with the heme propionate group resulted in the elevation of the heme potential of this mutant, and therefore, FMN to heme iron electron transfer was accelerated [70].

A similar interaction with the heme propionate group was shown in the I401E variant with the Glu401 residue sharing a water-mediated bond with the heme propionate (Figure 10C). The glutamate residue was surrounded by many water molecules and shared multiple hydrogen bonds with these water molecules. This resulted in an altered heme environment coupled with a more positive heme potential compared to the wild-type enzyme and the fastest heme reduction rate compared to the wild type and mutants (Figure 10C). Glu401 was located further away from the heme and, therefore, no interactions were formed between the heme propionate group and the mutated residue (Figure 10C). This residue interacted with two water molecules that are not normally present within the CYP102 active site (Figure 10C) [70].

Overall, all variant enzymes lacked the covalent linkage between the heme methyl group and the glutamate residue [70]. However, these mutations revealed the effects on the thermodynamic and substrate binding of CYP102. The L86E variant showed a higher binding affinity for laurate and arachidonate substrates compared to the wild-type enzyme, and the introduction of new water molecules close to the heme in the F261E variant may affect the electrostatic environment [70]. The results indicate that the glutamate–heme interaction seen in CYP4 enzymes is disfavored in CYP102, but the introduction of an acidic residue stabilized the heme complex and the heme environment.

#### 2.4.6. Pentuple Mutant of CYP102 Highlights a Possible Mechanism for the Conformational Shift of CYP102

**Figure 11:**
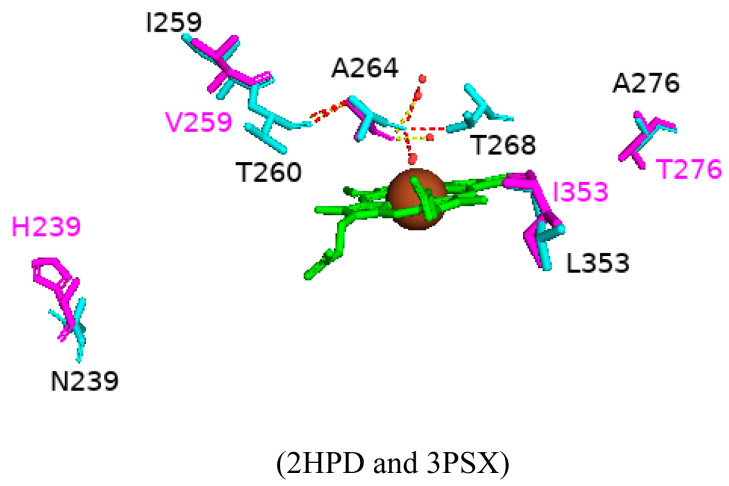
Analysis of active sites of wild-type and pentuple mutant of CYP102. The active sites of wild-type and pentuple mutant of CYP102 are superimposed. Heme is shown in green; the iron molecule is shown in brown. Wild-type amino acid residues that share a polar interaction are shown in cyan, and mutated amino acids are shown in magenta. Polar interactions are indicated as red dashed lines for the wild type and yellow for the mutant; water molecules are represented as red dots, and amino acid residues are labeled according to their single-letter codes. PDB code is shown underneath the respective model.

Many variants of CYP102 have been designed to incorporate amino acid mutations either remotely or close to the active site. However, these variants do not have a substrate with a C-H bond near the heme iron. A CYP102 pentuple mutant (A191T/N239H/I259V/A276T/L353I) of amino acid residues that have no specific structural importance was designed in order to understand the impacts of these substitutions further [71]. The A191T mutation was not resolved in the enzyme crystal structure and thus did not appear in the protein model (Figure 11). The other substitutions led to the reorganization of the G and I helices and caused many conformation changes to the enzyme whereby it resembled the substrate-bound structure. Examples include reduction of the kink seen in the I helix that is associated with dioxygen activation [71]. Unusually, the sixth water ligand is hydrogen bonded to the carbonyl oxygen of Ala264, as the axial water ligand moved to this “alternative” site due to the substrate-induced conformation change (Figure 11). Repositioning the I helix may have led to the sixth water ligand remaining ligated to the heme iron instead of displacing it to the alternative location [71]. However, the five mutations cannot explain these conformation changes within this enzyme. Despite the unresolved impact of the threonine residue in mutant A191T, the amino acid residue is located in the F/G loop, a highly dynamic area of CYP102. Therefore, introducing a polar amino acid residue may alter the environment of the F/G loop and result in the G and I helices’ movement as there is no significant change in the secondary structure of the protein around the other mutations [71]. These results highlight the consequences of secondary structure motion and its overall effect on the conformational shift of CYP102.

#### 2.4.7. Effects of Mutations Further Away from the Active Site on the Conformation Equilibrium of CYP102

**Figure 12:**
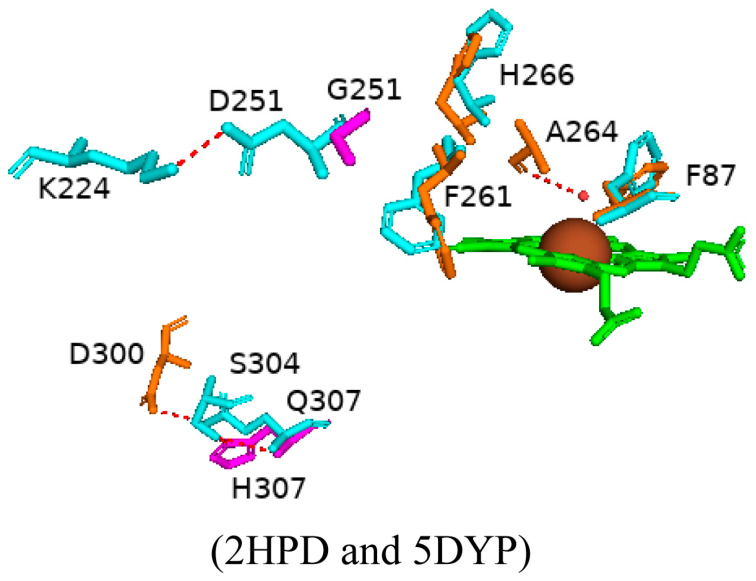
Analysis of CYP102 active sites of wild-type and double mutant enzymes (D251G and Q307H). The active sites of wild type and double mutant are superimposed. Heme is shown in green; the iron molecule is shown in brown. Wild-type amino acid residues are shown in cyan, mutated amino acids in magenta, and amino acids within the mutant are shown in orange. Polar interactions are indicated as red dashed lines; water molecules are represented as red dots, and amino acid residues are labeled according to their single-letter codes. PDB code is shown underneath the respective model.

CYP102 random mutagenesis of two amino acid residues distant from the active site resulted in a double mutant (D251G and Q307H) that showed promising activity towards generating metabolites of diclofenac, ibuprofen, and tolbutamide [33]. Structural analysis of this variant reveals major changes in the F and G helices, which compose the substrate CYP102 access channel. The G helix shows a higher degree of flexibility, which may be a result of the D251G substitution. Further, the introduction of glycine in this region breaks the salt bridge between Asp251 and Lys224, causing the G helix to be repositioned (Figure 12) [72]. The substitution of histidine instead of glutamine on the protein surface replaces, not abolishes, the hydrogen bond between Gln307 and Asp300 with Ser304, so the structure is not drastically altered compared to the D251G mutation (Figure 12).

There are many structural aspects of this variant that suggest it may be adopting the closed conformation of CYP102. The sixth water ligand is slightly displaced from the heme iron and retains its hydrogen bond to Ala264 (Figure 12). The I-helix kink is less pronounced, which is associated with the closed conformation of CYP102. Phe87 is known to rotate upon substrate binding in the wild-type enzyme, and this residue is almost completely rotated in the variant (Figure 12). Phe261 and His266 are located in close proximity to the heme iron and are known to undergo major conformational changes upon substrate binding, and these residues adopt their closed conformation positions in the double variant (Figure 12) [72]. Therefore, the variant enzyme adopts the closed conformation as seen for wild-type CYP102 in the absence of a substrate, as has also been noticed in many other variants. However, the conformational shift was due to mutations near the active site, whereas there is only one mutation on the protein surface that breaks a salt bridge resulting in the conformational shift in this variant. These results indicate that a mutation far away from the active site can influence CYP102 conformational equilibrium via flexibility of the substrate access channel.

#### 2.4.8. Ten Mutations of CYP102 Highlight the Relationship Between the High Flexibility of Certain Regions and Substrate Specificity

**Figure 13:**
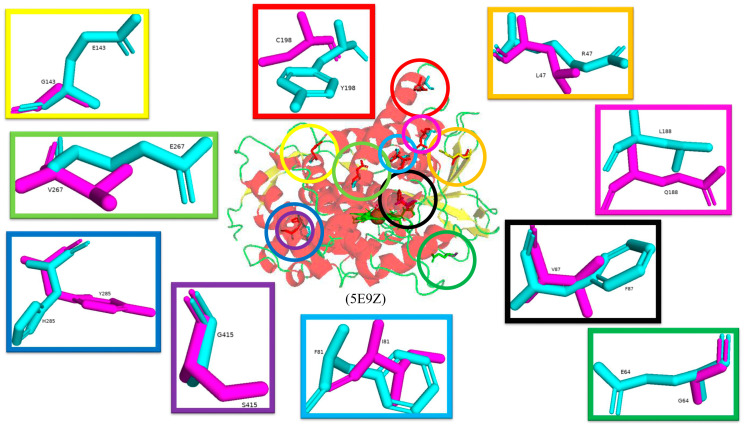
Schematic diagram representing CYP102 variant containing ten mutations (R47L, E64G, F81I, F87V, E143G, L188Q, Y198C, E267V, H285Y, and G415S). The overall structure of mutated CYP102 is shown in the middle, where wild-type amino acid residues are shown in cyan, and mutated amino acids are shown in magenta. The position of each mutated amino acid is shown within a colored circle, and the conformation of the wild type and mutated amino acid is shown in a box with the corresponding color. Alpha helices are red ribbons; beta sheets are yellow arrows; loops are green lines, and amino acid residues are labeled according to their single-letter codes. Heme is shown in green; the iron molecule is shown in brown. PDB code is shown underneath the respective model.

Random mutagenesis of amino acid residues within CYP102 is a highly common approach to engineering this enzyme due to its high catalytic turnover rate and biotechnological importance. A combination of all the mutations that led to an improved CYP102 catalytic enzyme resulted in a variant protein that has ten mutations, namely, R47L, E64G, F81I, F87V, E143G, L188Q, Y198C, E267V, H285Y, and G415S (Figure 13) [73]. This variant is known for selectively producing metabolites of non-steroid anti-inflammatory drugs such as mefenamic acid [73]. The crystal structure of this variant provides critical information on the role of specific amino acid residues in determining the unique selectivity of this variant. Similarly to other CYP102 variants, this variant adopts the closed conformation in the absence of a substrate, which is identified by the movement of the F and G helices, resulting in the closure of the substrate access channel and the kink in the I helix being diminished.

Arg47 is located near the entrance of the CYP102 substrate access channel; substitution to leucine caused a slight tilt in the beta strands, permitting the lipophilic side to be buried (Figure 13) [73]. Within the F helix, the substitution of glutamine does not affect the architecture of the substrate access channel (Figure 13). The region that interacts with the FMN-binding domain contains the next mutation of E46G and the hydrogen bond between glutamic acid and glutamine is disrupted and could affect the interactions between the heme and the FMN-binding domain [73]. Helix B’ contains the next mutation, F81I, and the lipophilic isoleucine side chain is embedded in the cavity previously occupied by phenylalanine in the wild type, resulting in a slight bend in the helix (Figure 13) [73]. Phe87 is situated near the heme iron, and many variants have this residue mutated to smaller sized amino acids in order to increase the space available for substrates to access the heme iron. The mutation to valine served the same purpose and increased the space available for substrates to access the reactive heme iron (Figure 13).

In CYP102, the I helix is known for its kink and contains the next mutation involving Glu267. In the wild-type enzyme, this residue forms a strong ionic bond with K440, which is disturbed by the substitution to valine. The cavity between helices E and F and the adjacent beta-strand are thus larger in the mutant enzyme (Figure 13). The G helix contains Tyr198, which does not have any specific interactions in the wild-type enzyme and, therefore, substitution to cysteine does not have any significant effects on the secondary structure of the variant enzyme (Figure 13) [73]. Similarly, E143G and H285Y, situated in helices E and J, respectively, are exposed to the solvent and do not have any significant effects on the variant (Figure 13). The secondary structure was also protected from any significant movements in the L helix, which contained the G415S mutation, and the result was no significant changes (Figure 13). Despite the multiple mutations within this variant, no significant conformational changes were observed besides two mutations that resulted in the breakage of strong interactions with other amino acid residues and led to the increased flexibility of those regions [73]. High helix flexibility can be associated with low substrate specificity, explaining this variant’s broad range of substrate selectivity.

**Figure 14:**
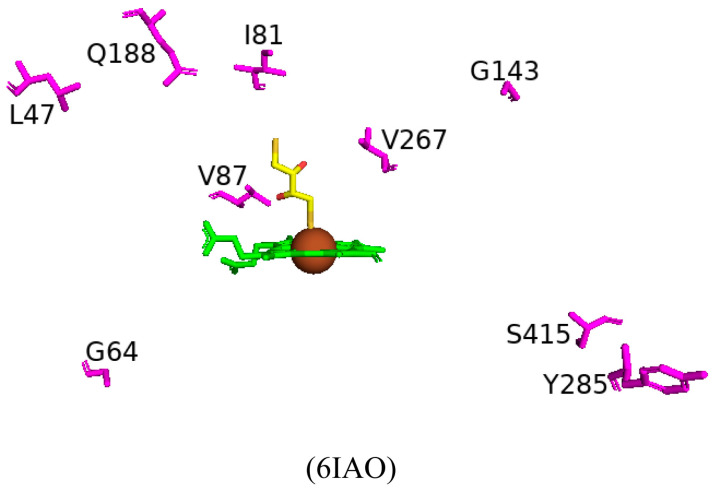
A variant of CYP102 bound with dithiothreitol. Heme is shown in green; the ligand is colored with carbon atoms as yellow, sulfur as light brown, oxygen as red, and the iron molecule is shown in brown. Mutated amino acids are shown in magenta, and amino acid residues are labeled according to their single-letter codes. PDB code is shown underneath the respective model. Amino acid residues within 5 Å of the ligand are shown in Table S2.

To test the broad range of substrate selectivity, the variant co-crystallized with dithiothreitol (DTT); the structure showed that the DTT molecule directly coordinated to the heme iron via its sulfur atom and the enzyme adopted a closed conformation (Figure 14) [74]. There were no direct hydrogen bonds shared between the ligand and the variant, and the binding of DTT did not significantly alter the protein conformation (Figure 14) [74]. The binding and orientation of DTT within the variant’s active site provide evidence that this variant can be considered a promiscuous enzyme for molecular modeling purposes.

### 2.5. Improving CYP102 Activity via Mutations Affecting Substrate Binding and Its Orientation

#### 2.5.1. A82F Variant Preserves the Hydrophobic Pocket in the Active Site and Leads to Tighter Substrate Binding

**Figure 15:**
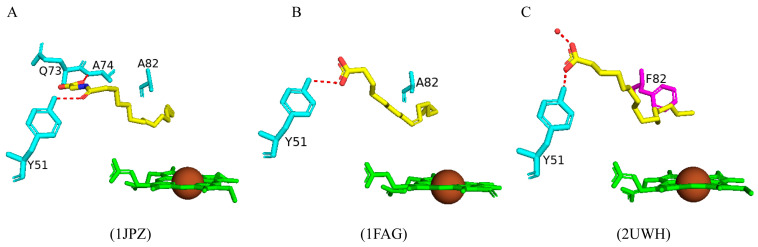
Analysis of CYP102 and its A82F variant interactions with different ligands. Wild-type enzyme bound with palmitate (**A**), wild-type enzyme bound with palmitoleic acid (**B**), and variant enzyme bound with palmitoglycine (**C**). Heme is shown in green; substrates are colored with carbon atoms as yellow, nitrogen as blue, and oxygen as red; the iron molecule is shown in brown. Wild-type amino acid residues that share a polar interaction are shown in cyan, and mutated amino acid is shown in magenta. Polar interactions are indicated as red dashed lines; water molecules are represented as red dots, and amino acid residues are labeled according to their single-letter codes. PDB code is shown underneath the respective model. Amino acid residues within 5 Å of the ligand are shown in Table S2.

The A82F variant was crystallized bound with palmitate and had a very similar structure when compared to the wild-type enzyme bound with palmitoglycine and palmitoleic acid, respectively (Figure 15A,B). The main difference noticed was the substrate orientation within the active site pocket (Figure 15C). The mutant enzyme Phe82 residue filled the hydrophobic pocket instead of destroying it as previously predicted [75]. As a consequence, the shape of the substrate binding channel changes and decreases in length, therefore causing the substrate to adopt a different binding orientation whereby the carboxylate group is closer to the protein surface (Figure 15C). The carboxylate group forms a direct hydrogen bond with Tyr51, as shown in the wild-type enzyme bound with palmitoleic acid (Figure 15B). The methyl group of the substrate points away and is almost parallel to the heme iron; despite the change in the binding position, the substrate still binds far away from the heme iron and adopts an inactive state of the enzyme (Figure 15C).

The A82F variant enzyme showed a higher affinity for the substrate and a greater conversion of the spin state of the heme iron as well as increased catalytic efficiency [75]. Despite the substrate binding far away from the heme iron, these results indicate that substituting the alanine residue for a bulkier amino acid residue such as phenylalanine causes a conformational shift to the substrate-bound form, thus increasing the substrate-binding affinity and resulting in tighter binding and a high-spin state of the heme iron.

#### 2.5.2. The A328V Variant Increases the Affinity for Hydrophobic Substrates and Results in Higher Binding Affinity

**Figure 16:**
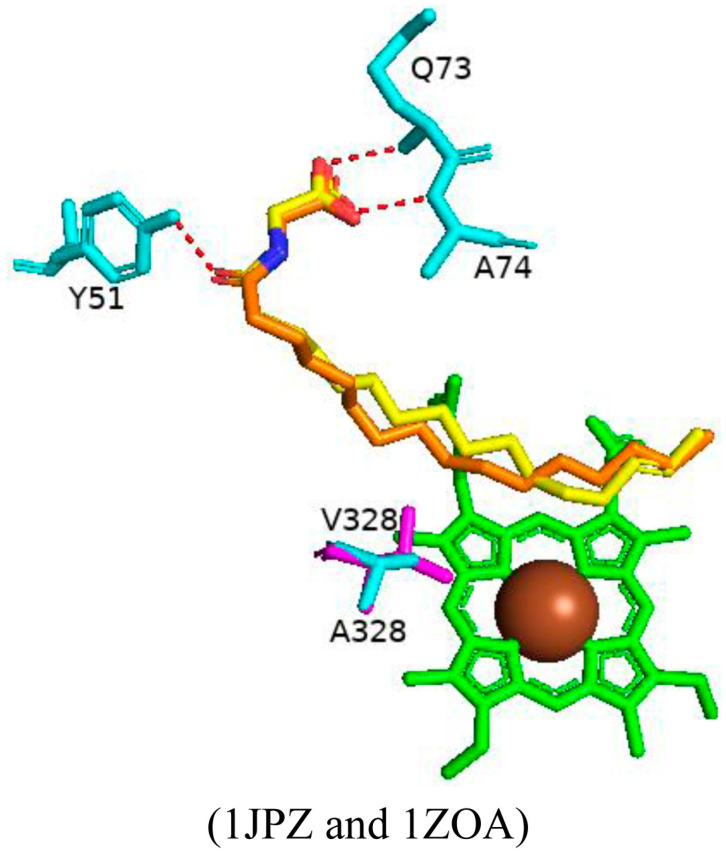
Comparative structural analysis of active site cavity of CYP102 (1JPZ) and its A328V (1ZOA) variant bound with palmitoglycine. The active sites of wild-type and double mutant enzymes are superimposed. Heme is shown in green; iron molecule is shown in brown, substrates bound to wild type and variant are colored with carbon atoms as yellow, nitrogen as blue, and oxygen as red and carbon atoms as orange, nitrogen as blue, and oxygen as red, respectively. Wild-type amino acid residues that share a polar interaction are shown in cyan, and mutated amino acids are shown in magenta. Polar interactions are indicated as red dashed lines, and amino acid residues are labeled according to their single-letter codes. PDB codes are shown underneath the respective model. Amino acid residues within 5 Å of the ligands are shown in Table S2.

Ala328 is in close proximity to the heme iron with the methyl side chain adjacent to the heme iron. Substitution of this residue may affect substrate specificity and, consequently, valine was substituted in this position to test this hypothesis [76]. The variant bound palmitoglycine and was predominantly in the high-spin state adopting a substrate-bound form of CYP102 like the wild type. Superimposition of the wild-type enzyme and variant structures revealed no significant architectural differences (Figure 16). The substrate in the variant enzyme shares identical polar interactions compared to the wild-type protein (Figure 16). A slight difference is noticed in the substrate orientation near the mutated amino acid; due to steric conflicts, the substrate moves slightly away to accommodate the valine methyl group (Figure 16) [76]. Besides this slight movement, no other striking differences were noticed, and therefore, the addition of two methyl groups was tolerated by the variant enzyme. Interestingly, the variant showed a slightly higher substrate-binding affinity and product formation rate was comparable to the wild-type enzyme, possibly due to the increased affinity to hydrophobic substrates and increasing the rate at which the enzyme–substrate complex is formed [76].

#### 2.5.3. CYP102 Variants Serve as Excellent Candidates for Magnetic Resonance Imaging Sensors

**Figure 17:**
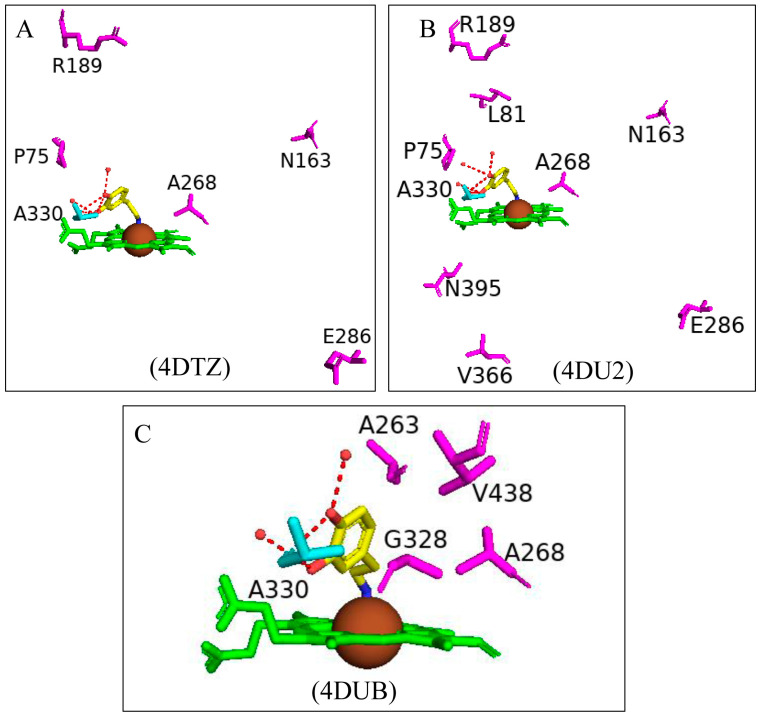
Active site cavity analysis of CYP102 variant enzymes bound with dopamine. Variant 1 (**A**), variant 2 (**B**), variant 3 (**C**). Heme is shown in green; the iron molecule is shown in brown; the substrate is colored with carbon atoms as yellow, nitrogen as blue, and oxygen as red. Wild-type amino acid residues that share a polar interaction are shown in cyan, and mutated amino acids are shown in magenta. Polar interactions are indicated as red dashed lines, and amino acid residues are labeled according to their single-letter codes. PDB code is shown underneath the respective model. Amino acid residues within 5 Å of the ligand are shown in Table S2.

Paramagnetic metalloproteins can be efficient magnetic resonance imaging (MRI) sensors due to the associated substrate selectivity and the ability to use site-directed mutagenesis to fine-tune the protein’s properties [40]. CYP102 is a prime candidate to start MRI sensor design as mutagenesis to increase the protein’s stability and specificity is highly common. Three variants with different mutations were designed and crystallized in the presence of dopamine. Variant 1 had five mutations (L75P, I163A, Q189R, T268A, and V286E), variant 2 had eight mutations (L75P, F81L, I163A, Q189R, T268A, V286E, Y395H, and I366V), and variant 3 had four mutations (I263A, T268A, A328G, and T438V) (Figure 17) [40]. All variants revealed direct coordination between the dopamine amine group and the heme iron (Figure 17).

**Figure 18:**
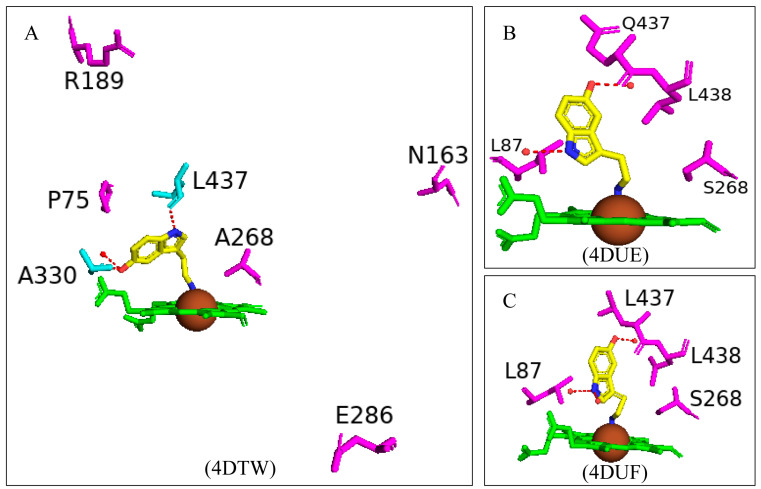
Active site cavity analysis of CYP102 variants bound with serotonin. Variant 1 (**A**), variant 4 (**B**), variant 5 (**C**). Heme is shown in green; the iron molecule is shown in brown; the substrate is colored with carbon atoms as yellow, nitrogen as blue, and oxygen as red. Wild-type amino acid residues that share a polar interaction are shown in cyan, and mutated amino acids are shown in magenta. Polar interactions are indicated as red dashed lines, and amino acid residues are labeled according to their single-letter codes. PDB code is shown underneath the respective model. Amino acid residues within 5 Å of the ligand are shown in Table S2.

Variants 1 and 2 share many common characteristics, such as the carbonyl group of Ala330 sharing a bifurcated hydrogen bond with the dopamine hydroxyl groups (Figure 18A,B). The dopamine molecule is stabilized in the active sites by two water molecule networks that form hydrogen bonds with the hydroxyl groups of the dopamine molecule (Figure 17A,B). The interactions between the dopamine molecule and the CYP102 variant enzyme are not related to the amino acid mutations [40]. These two variants share three active site mutations, namely, L75P, I263A, and T268A (Figure 17A,B). In the wild type, Thr268 is found within the I helix and shares a direct hydrogen bond with Ala264 causing a kink in the I helix. This kink is still present in the variant despite the mutation to alanine. However, the I helix is slightly rotated causing an unfavorable interaction with Ile263 which in these variants is mutated to alanine, alleviating the steric clash [40]. The smaller amino acid residue may also alleviate the steric hindrance between Thr268 and the amine group of dopamine. Substitution to proline in the L75P mutation improves the hydrophobicity of the environment surrounding the dopamine molecule [40]. This residue is located in the B’ helix and its mutation does not affect the protein structure in variant 1. However, in variant 2, mutation of another B’ helix residue, F81L, causes the collapse of this helix which may also be a consequence of other mutations [40]. The hydrogen-bonding network between the hydroxyl groups increases the binding affinity of the molecule. However, the increased van der Waals forces from the side chain mutations influence the increased affinity more than the hydrogen bonding alone.

Variant 3 showed an improved ligand affinity and selectivity compared to the other two variants [40]. The major difference between the other variants and variant 3 is the orientation of the dopamine molecule in the active site. Because of the A328G mutation, the dopamine molecule is permitted to rotate and adopts a more favorable orientation, being slightly tilted (Figure 17C). There are no differences in the hydrogen-bonding patterns in all variants, and therefore, the increased binding affinity may be due to the improved van der Waals interactions surrounding the dopamine catechol ring.

Variant 1 showed an increased affinity towards serotonin and variants 4 and 5 were designed to investigate the enzyme affinity towards dopamine and serotonin. Interestingly there were no improvements in the binding affinity towards dopamine although there was an increased affinity towards serotonin [40]. Variant 4 contained four mutations (F87L, T268S, L437Q, and T438L) and variant 5 had three mutations (F87L, T268S, and T438L) (Figure 17). All mutations did not result in major changes in the protein stability [40]. In a similar fashion to dopamine, the amine group of serotonin directly coordinates with the heme iron but does not interact with the carbonyl group of Ala330 besides variant 1 (Figure 18). The serotonin molecule is oriented with the hydroxyindole group facing the two amino acid mutations and the hydroxyl group shares a hydrogen bond with a water molecule (Gln437 and Leu438). This conformation is energetically favorable and significantly increases the affinity toward serotonin [40].

The increased serotonin-binding affinity may be in part due to the favorable aliphatic hydrophobic amino acid, leucine, compared to phenylalanine at the 87th position. The hydroxyindole ring is slightly rotated in variant 4 when compared to variant 5 and this may be due to the L437Q orientation, and the amide moiety adopts an orientation that is favorable for a dipole–dipole interaction (Figure 18C) [40]. The increased hydrophobic interactions and hydrogen bonding to water networks may increase the binding affinity in these variants. It is very interesting to note that variants 4 and 5 had a higher substrate selectivity towards serotonin instead of dopamine whereas variants 1 and 2 had similar selectivity towards both substrates [40]. The structure of dopamine is unfavorable for variants 4 and 5 as it is shorter compared to serotonin and therefore would not experience the same favorable hydrophobic packing, lacking the indole nitrogen and not able to form hydrogen bonds with the water networks [40]. CYP102 has the impressive ability to accept amphipathic substrates within its active site, considering that the majority of its substrates are long-chain aliphatic fatty acids. Therefore, it is shown that a few active site mutations greatly increased the variants’ affinity towards dopamine and serotonin. This proves that site-directed mutagenesis can be used as a tool to significantly improve CYP102’s performance as a ligand-responsive MRI sensor.

#### 2.5.4. A Double Mutant of CYP102 Shows Promising Interactions with Omeprazole and Azole Drugs

**Figure 19:**
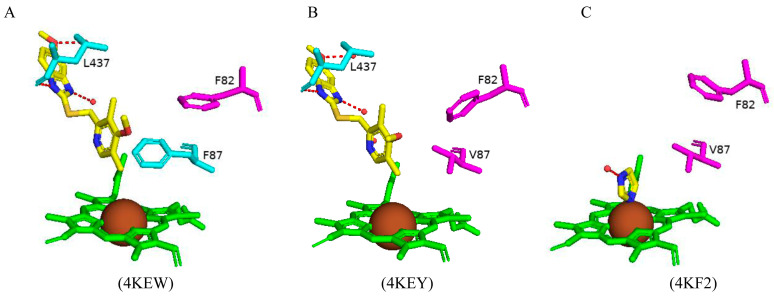
Active site analysis of CYP102 variants bound to different drugs. Variant 1 bound with omeprazole (**A**), variant 2 bound with omeprazole (**B**), variant 2 bound with imidazole (**C**). Heme is shown in green; the iron molecule is shown in brown; the substrate is colored with carbon atoms as yellow, nitrogen as blue, and oxygen as red. Wild-type amino acid residues that share a polar interaction are shown in cyan, and mutated amino acids are shown in magenta. Polar interactions are indicated as red dashed lines, and amino acid residues are labeled according to their single-letter codes. PDB codes are shown underneath the respective model. Amino acid residues within 5 Å of the ligands are shown in Table S2.

Cytochrome P450s are well known for their regio- and stereoselectivity, an attribute that is very attractive in the biotechnological production of oxychemicals. Omeprazole is a proton pump inhibitor and is hydroxylated to 5-hydroxy omeprazole by human P450, CYP2C19 [77]. Oxidized metabolites of human drugs are crucial in the pharmaceutical industry. CYP102 is a great model enzyme to generate metabolites in large quantities in order to examine these metabolites produced by human P450s. Mutations of CYP102 active site residues have revealed many amino acid residues that affect substrate specificity and affinity. For example, Phe87 mutations have been shown to alter the regioselectivity of fatty acid oxidation and Ala82 mutations have been shown to greatly increase the enzyme’s affinity for fatty acid substrates [32]. Using this knowledge, two variants were designed incorporating these mutations and tested against omeprazole. Variant 1 had one mutation (A82F), and variant 2 had two mutations (A82F and F87V) (Figure 19A,B). The crystal structure of both variants reveals the pyridinyl moiety of the omeprazole substrate to be almost perpendicular to the heme iron; the substrate is also bound in close proximity to the heme iron with the methyl group near the heme iron, resulting in displacement of the sixth water ligand (Figure 19A,B). The methyl group is identified as the hydroxylation site during catalysis and studies have revealed that 5-hydroxy omeprazole was produced by these variants [32].

There is a slight difference in the substrate orientation between the two variants and this may be due to the F87V mutation in variant 2. This substitution increases the available space and allows for the substrate to adopt a less strained conformation, explaining the higher binding affinity observed for this variant (Figure 19B). In variant 1, the Phe87 residue is in close contact with the pyridinyl moiety, and to reduce steric conflicts, there is a slight bend in the substrate which is absent in variant 2 (Figure 19A,B). The substrate shares a direct hydrogen bond with Leu437 and one of the methoxybenzimidazole nitrogens, whilst the other nitrogen atom and hydroxyl group also interact with water molecules (Figure 19A,B). It is interesting to note that in the crystal structure of the substrate-free form of variant 1, an imidazole molecule that was present in the crystallization buffer cocrystallized with direct coordination with the heme iron (Figure 19C) [32]. This provides crucial evidence that the A82F mutation leads to significant changes in the structure of the substrate-free form of the variant. The drastic changes are seen in the positioning of the F and G helices which adopts a substrate-bound conformation and increases the variant mobility, improving ligand-binding ability [32]. This repositioning is the result of a wave of movements starting with steric conflicts between Ile263 and Phe82 resulting in a shift of the I helix, which leads to a shift in the G helix and is accompanied by reorientation of the hydrophobic residues in the B and F helices causing the F and G helices to move further away [32].

The shift from open to closed conformation of CYP102 upon substrate binding is highly dependent on the free energy difference of both conformational states and ligand binding [32]. The A82F mutation may destabilize the substrate-free conformation and favor the substrate-bound form. The structural changes induced by this single mutation highlight its role as a gatekeeper residue for preferred substrate access as a result of its impact on the regulation of CYP102 conformation equilibrium [32]. The A82F mutation enabled the tight binding of omeprazole and permitted the same hydroxylation reaction catalyzed by CYP2C19 [77]. The combination of both mutations regulates the conformational equilibrium and alters the environment around the heme iron, causing significant changes to substrate selectivity, which is advantageous for biotechnological applications.

**Figure 20:**
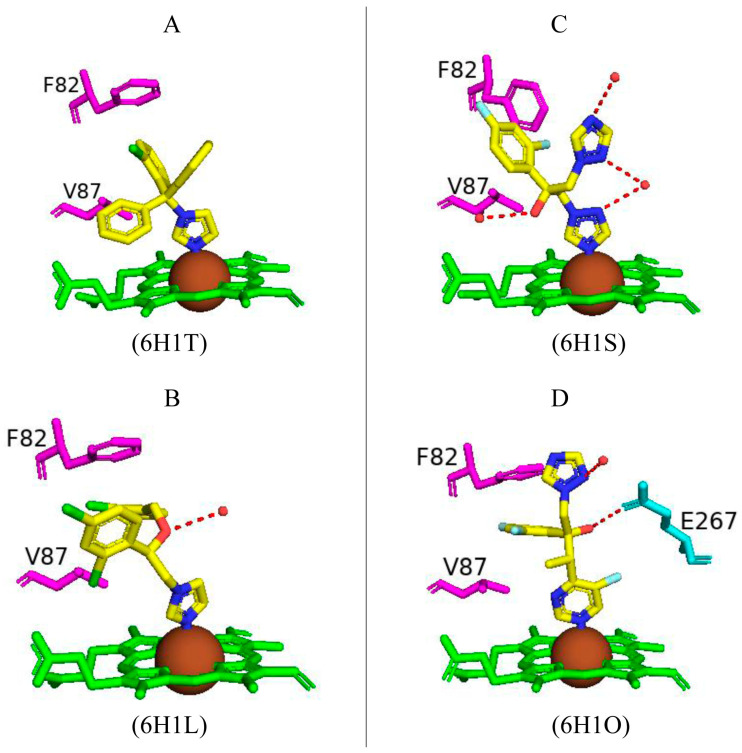
Active site analysis of CYP102 double mutants bound to different azole drugs. Double mutant bound with clotrimazole (**A**), tioconazole (**B**), fluconazole (**C**), voriconazole (**D**). Heme is shown in green; iron molecule is shown in brown; ligand is colored with carbon atoms as yellow, nitrogen as blue, chlorine as green, fluorine as light blue, and oxygen as red. Wild-type amino acid residues sharing a polar interaction are shown in cyan, and mutated amino acids are shown in magenta. Polar interactions are indicated as red dashed lines; water molecules are shown as red dots, and amino acid residues are labeled according to their single-letter codes. PDB codes are shown underneath the respective model. Amino acid residues within 5 Å of the ligands are shown in Table S2.

Whilst CYP102 wild-type enzyme does not efficiently bind many azole drugs, the double mutant (A82F and F87V) bound four azole drugs, two imidazoles (clotrimazole and tioconazole) and two triazoles (fluconazole and voriconazole) (Figure 20) [78]. Azole drugs are known to directly coordinate with the heme iron of many P450s via a nitrogen atom in the imidazole or triazole ring [54]. Interestingly, all four azole drugs were bound within the active site of this variant, indicating the increased flexibility (Figure 20) [78]. All azole drugs bound to the variant tighter and with a higher binding affinity compared to the wild-type enzyme, a consequence of the combination of both a larger active site and structural rearrangements induced by the mutations [78]. It is evident that the conformational flexibility of the double mutant is an important aspect of the enzyme to ensure the complete binding of different ligands.

#### 2.5.5. Mutations of Amino Acid Residues Within the B’ Helix, F Helix, and β-Sheet 4 of CYP102 Influence the Protein’s Stability

**Figure 21:**
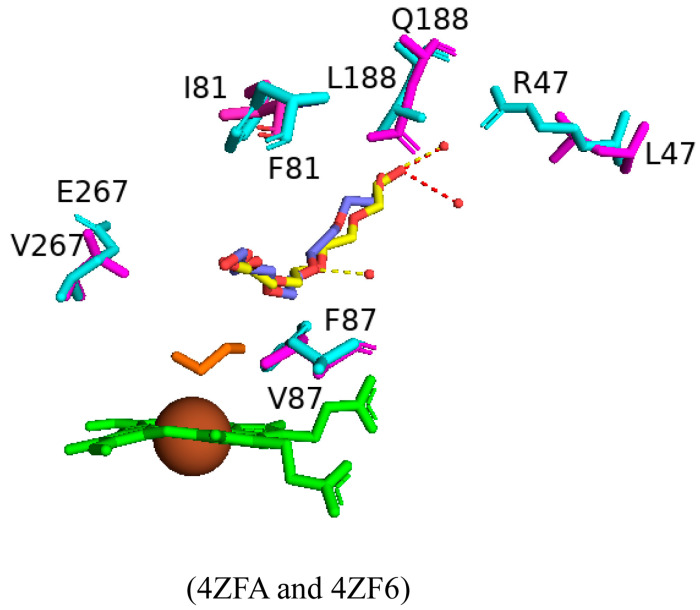
Active site superimposition of CYP102 and its pentuple mutant. Heme is shown in green; iron molecule is shown in brown; ethylene glycol is shown in orange; poly-ethylene glycol substrate is colored with carbon atoms as yellow and oxygen as red and carbon atoms as blue and oxygen as red for CYP102 and its pentuple mutant. Wild-type amino acid residues are shown in cyan, and mutated amino acids are shown in magenta. Wild-type polar interactions are indicated as red dashed lines; mutant polar interactions are indicated as yellow dashed lines; water molecules are shown as red dots, and amino acid residues are labeled according to their single-letter codes. PDB codes are shown underneath the respective model. Amino acid residues within 5 Å of the ligands are shown in Table S2.

Multiple CYP102 variants have been designed and probed for various novel functional roles. Despite increased substrate binding and specificity observed by these variants, the variants often exhibit low protein stability compared to the wild-type enzyme, impeding their industrial application [79]. A protein’s stability is measured by kinetic and thermodynamic stability; increased resistance to protein unfolding will likely enhance the kinetic stability of the protein [79]. Therefore, the wild-type and pentuple variant CYP102 enzymes were subjected to pulse proteolysis to analyze the protein’s stability [79]. The pentuple mutations included R47L, F81I, F87V, L188Q, and E267V (Figure 21).

Superimposition of the wild-type and the pentuple variant revealed a polyethylene glycol (PEG) molecule bound in the substrate access channel, which explains why the variant was found in a closed conformation (Figure 21). The mutations led to differences in the variant, and the substitution of a smaller amino acid in the R47L mutation creates a cavity which the adjacent amino acids fill and they disrupt hydrophobic interactions. Similarly, the E267V mutation disrupts the interaction between Thr438 and Lys440 [79]. The F87V mutation increases the available space near the heme iron, and, compared to the wild-type enzyme, an ethylene glycol molecule is bound in close proximity to the heme iron (Figure 21).

**Figure 22:**
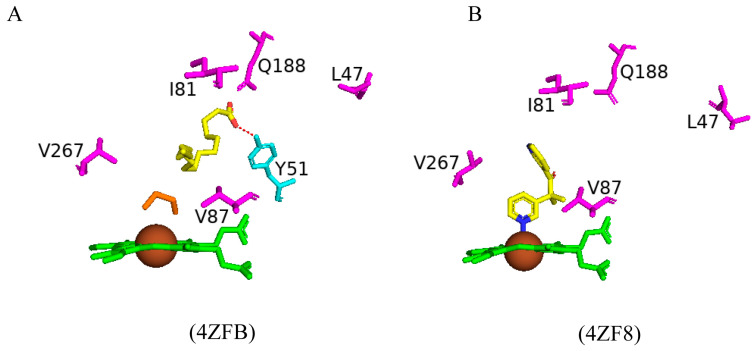
Active site cavity analysis of CYP102 pentuple mutant enzyme bound with palmitic acid (**A**), and metyrapone (**B**). Heme is shown in green; iron molecule is shown in brown; ethylene glycol is shown in orange; ligands are colored with carbon atoms as yellow, nitrogen as blue, and oxygen as red and mutated amino acids are shown in magenta. Amino acid residues are labeled according to their single-letter codes. PDB codes are shown underneath the respective model. Amino acid residues within 5 Å of the ligands are shown in Table S2.

To probe the stability of the protein–substrate complex the pentuple variant was crystallized with palmitic acid and an inhibitor, metyrapone (Figure 22A,B). Palmitic acid bound within the active site in a “folded” conformation, with the substrate stabilized by a hydrogen bond formed between the carboxylate group and Tyr51 and by hydrophobic interactions with Gln188 (Figure 22A). The F87V mutation increases the size of the hydrophobic pocket and accommodates the hydrophobic tail of the fatty acid. The increase in space also permits an ethylene glycol molecule to occupy the space close to the heme iron (Figure 22A) [79]. Metyrapone inhibits many P450s via direct coordination of its pyridine nitrogen atom to the heme iron, preventing the enzyme from oxidizing its substrate [79]. This same interaction is observed between the pentuple variant and metyrapone, and the binding of this inhibitor is assisted by the F87V mutation as Phe87 collides with metyrapone and prevents direct coordination with the heme iron (Figure 22B) [79]. Comparison of the mutated amino acids’ orientations in the pentuple mutant with palmitic acid and metyrapone revealed no major differences in all mutated residues except for Leu47, which relocates to a solvent-exposed position. This may be due to the lack of a substrate being part of the substrate access channel, inducing more flexibility within this region (Figure 22).

Pulse proteolysis experiments indicated that the pentuple variant was less stable than the wild-type enzyme and pointed to the possibility that the mutations destabilize the enzyme [79]. The substitution of the non-polar amino acid residue leucine to the polar amino acid asparagine within the substrate access channel affected its interactions with Gln73 in the B’ helix and at the entrance of the channel, mutation of residue 188 in the F helix from the non-polar leucine to the polar asparagine impacts its interaction with Q73 in the B’ helix. Glu267 broke the salt bridge with Lys440 in β-sheet 4 and Ile81 in the B’ helix affected the hydrophobic interactions with the F, G, and I helices [79]. The mutations disrupted important salt bridges and hydrophobic interactions, resulting in a destabilised enzyme.

#### 2.5.6. Arg47 Plays a Role in the Binding and Positioning of N-Palmitoylglycine During the Initial Binding Step in the Mechanistic Reaction of CYP102

**Figure 23:**
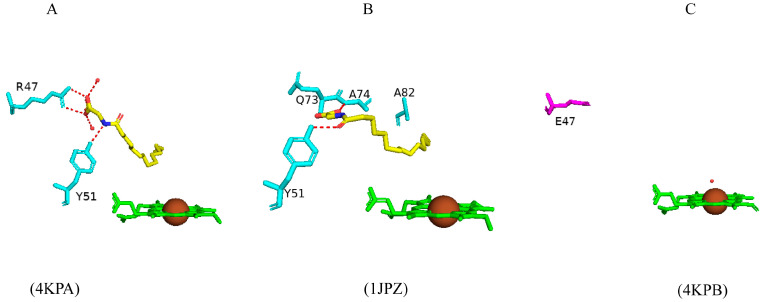
Active site structural analysis of CYP102 crystallized with N-palmitoylglycine at initial (**A**) and later stages (**B**) of catalytic activity and the variant CYP102 R47E (**C**). Heme is shown in green; the iron molecule is shown in brown; ligands are colored with carbon atoms as yellow, nitrogen as blue, and oxygen as red and mutated amino acid is shown in magenta. Polar interactions are shown as red dashed lines; water molecules are shown as red dots, and amino acid residues are labeled according to their single-letter codes. PDB codes are shown underneath the respective model. Amino acid residues within 5 Å of the ligands are shown in Table S2.

Understanding CYP102–substrate complex binding and dynamics is a hot topic, as the majority of the ligands are bound far away from the heme iron in an inactive state. Arg47 is the only charged residue of CYP102 that is located in the opening of the substrate access channel and it has been proposed to play an important role in initial substrate binding and assisting in adopting a correct orientation within the active site [23]. To probe the role(s) of this residue it was mutated to the oppositely charged residue, glutamic acid. The wild-type enzyme was crystallized with the substrate but interestingly was in a different space group compared to the first crystal structure bound to N-palmitoylglycine, indicating the substrate to be in a different conformation with Arg47 interacting with the carboxylate group via a direct bidendate ion pair (Figure 23A,B) [23]. The different substrate conformations between the two structures reveal possible dynamics of the initial binding steps of the carboxylate group [23]. The mutation R47E had the greatest impact on enzyme activity and substrate binding, and the crystal structure of this variant revealed the glutamic acid residue blocking the entrance of the binding pocket, resulting in a low binding affinity for N-palmitoylglycine, thus no substrate was bound in the active site (Figure 23C) [23]. Therefore, Arg47 is essential for the binding of N-palmitoylglycine during the initial binding steps.

#### 2.5.7. Improving the Enantioselectivity of CYP102 Towards Non-Native Substrates

**Figure 24:**
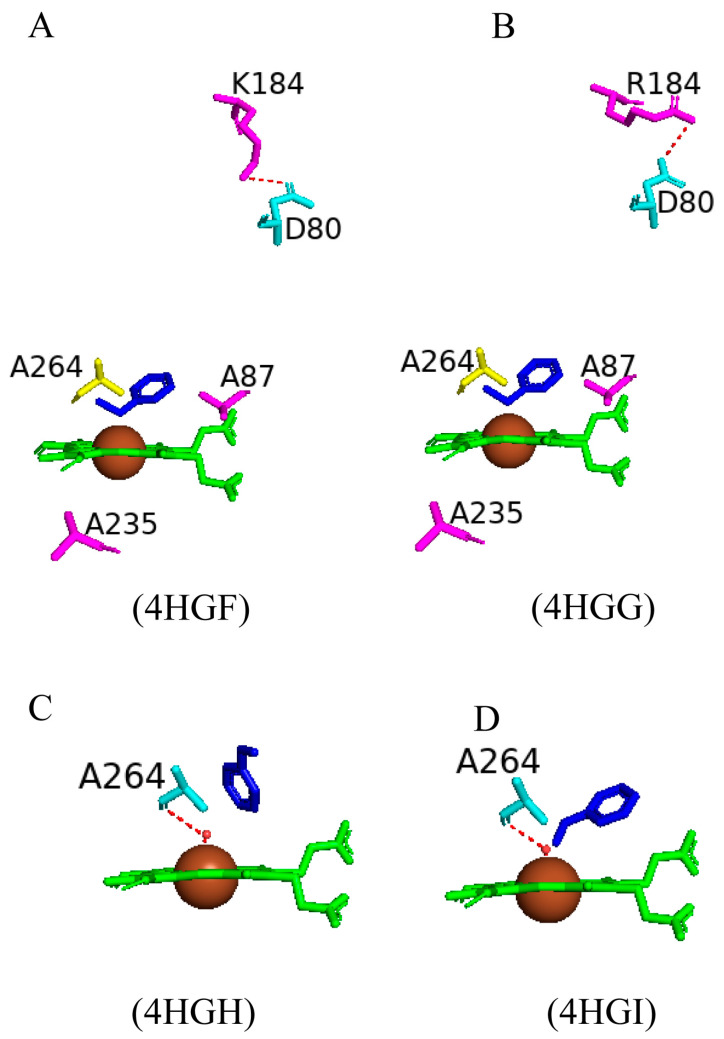
Active site cavity analysis of CYP102 variants bound with styrene. (**A**) CYP102 F87A, T235A, A184K with styrene; (**B**) CYP102 F87A, T235A, A184R with styrene; (**C**,**D**) parent variant (CYP102 F87A and T235A) with styrene after 2–3 min and after 60 min. The styrene molecule is shown in blue; heme is shown in green; the iron molecule is shown in brown; the amino acid discussed is shown in yellow; mutated amino acid residues are shown in magenta, and amino acid residues sharing a polar interaction are shown in cyan. Polar interactions are indicated as red dashed lines; water molecules are shown as red dots, and amino acid residues are labeled according to their single-letter codes. PDB code is shown underneath the respective model. Amino acid residues within 5 Å of the ligand are shown in Table S2.

Styrene oxide is well known as a versatile intermediate in the synthesis of valuable chemicals, including antidiabetic agents and dopamine agonists [80]. A CYP102 variant with two mutations close to the heme iron has shown high catalytic activity towards styrene, the mutations being F87A and T235A. This variant will be referred to as the “parent variant”. The addition of a single mutation of the Ala184 residue, further away from the active site, led to the inversion of the enantioselectivity of styrene epoxidation towards styrene oxide [80]. Two variants of the parent mutant (variant 1 with A184K and variant 2 with A184R) were designed, and styrene bound to the active site (Figure 24A,B). The mutation of Ala184 resulted in a salt bridge forming with Asp80, and this led to the F helix lifting and opening the substrate access channel, permitting access of the styrene molecule into the active site [80]. The change in F and G helices’ orientation in these variants resulted in the reorientation of the I helix N-terminal, and therefore, a water molecule perturbed the hydrogen-bonding network and the well-associated kink in the I helix was diminished, which led to Ala264 relocating further away from the active site and thus the pro-S faced towards the heme iron (Figure 24A,B) [80].

The parent variant of CYP102 was crystallized with styrene after different time intervals of soaking. Type 1 was soaked for 2–3 min, and type 2 was soaked for 60 min [80]. The type 1 crystal structure revealed the phenyl ring of styrene to be oriented towards the heme iron, and the sixth water ligand is coordinated to the heme iron sharing a direct hydrogen bond with Ala264 (Figure 24C). The vinyl tail is located further away from the heme iron, and therefore, epoxidation could not occur as this represented a non-productive state [80]. Styrene was oriented differently in type 2, with the vinyl tail located close to the heme iron and the pro-R pointing towards the heme iron. The sixth water ligand was oriented to the heme iron, and the hydrogen bond to Ala264 remained; this can be considered as the productive state of the enzyme (Figure 24D) [80]. The I helix in the parent variant is not disturbed and the kink is well-defined and this leads to the Ala264 residue providing steric conflicts with styrene and prevents the exposure of the pro-S to the heme iron resulting in the formation of R-styrene epoxide [80]. These results highlight the role that steric hindrances play in the enantioselectivity of the formation of S-styrene oxide.

### 2.6. Engineering CYP102 for Small-Molecule Oxidation in Combination with Decoy Molecules

#### 2.6.1. Perfluorinated Carboxylic Acids (PFCs)

Biocatalysts that can selectively hydroxylate less reactive gaseous alkanes are highly sought after in the industrial field. P450s are well-known for their ability to hydroxylate C-H bonds and, because their recombinant forms can be prepared in large quantities using *Escherichia coli*, they show a lot of promise for future engineering strategies and their use in synthetic chemistry [41]. However, P450s are highly substrate-specific and generally do not hydroxylate non-endogenous substrates. The substrate specificity of P450s stems from the intermolecular interactions between the substrate and the amino acid residues within the binding pocket [41]. Although the endogenous substrate of CYP102 is not known, CYP102 may follow the same pattern and does not efficiently catalyze non-native substrates. A few intermolecular interactions that are required to “switch on” CYP102 activity include hydrophobic interactions with the alkyl chain of fatty acid and hydrogen bonding between the carboxylate of the fatty acid and amino acid residues Arg47 and Tyr51 [41]. These interactions were seen between CYP102 and palmitoleic acid (Figure 3A). Interestingly, CYP102 bound with N-palmitoylglycine revealed additional hydrogen bond networks between the carboxylate and Gln73 and Ala74, possibly resulting in increased binding affinity (Figure 3B). Apart from these interactions, it is very important that the alkyl chain enters the active site first, as this results in the displacement of the sixth water ligand and thus initiates the P450 catalytic cycle [41].

Using this information, perfluorinated carboxylic acids (PFCs) were designed as decoy molecules for CYP102. Interestingly, CYP102 misidentifies the decoy molecule as a fatty acid and this leads to a shift of the iron valency from a low-spin state to a high-spin state [41]. This misrecognition was utilized to hydroxylate substrates such as propane and benzene [81]. However, these decoy molecules had a low binding affinity and catalytic activity, therefore an amino acid was added to the PFCs to increase the binding affinity to CYP102 via favorable interactions [41]. N-perfluoroacyl amino acids, possessing hydrophobic side chains, were designed as new decoy molecules to bind CYP102. Among these decoy molecules, N-perfluorononanoyl-L-leucine had a high binding affinity (Kd/μM = 39) and was the most effective for propane and ethane hydroxylation with the highest product formation rate (256/min/P450 and 45/min/P450), respectively, compared to other decoy molecules [41].

**Figure 25:**
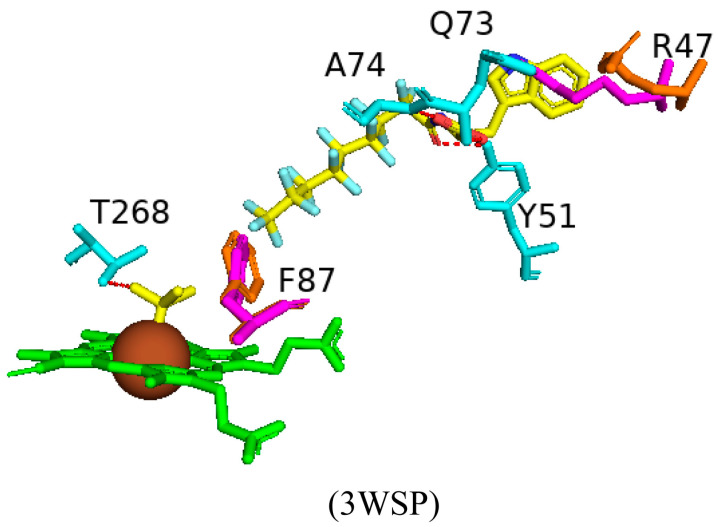
Active site cavity analysis of CYP102 bound with a decoy molecule, N-perfluorononanoyl-L-tryptophan, and dimethylsulfoxide (DMSO). The decoy molecule is colored with carbon atoms as yellow, nitrogen as blue, fluorine as light blue, and oxygen as red, heme is shown in green, the iron molecule is shown in brown, and the DMSO molecule is shown in yellow. Amino acid residues in the native closed conformation are shown in magenta and those in the non-native closed conformation are shown in orange. Polar interactions are indicated as red dashed lines, and amino acid residues are labeled according to their single-letter codes. PDB code is shown underneath the respective model. Amino acid residues within 5 Å of the ligand are shown in Table S2.

One short-chain N-perfluoroacyl amino acid containing tryptophan was bound and crystallized within CYP102 and shared direct hydrogen bonds with Tyr51, Gln73, and Ala74 (Figure 25). The sixth water ligand position was occupied by a DMSO molecule, and this provides evidence that small alkanes can enter the active site and bind close to the heme iron in the presence of this short-chain decoy molecule (Figure 25). The increased hydrophobic interactions between fluorine atoms and the substrate channel possibly led to the expulsion of water molecules from the active site cavity [41].

The orientation of two amino acid residues, in particular, provided further evidence that the introduction of the decoy molecule resulted in a shift to a partially closed conformation. Arg47 is located at the entrance of the substrate access channel and moves closer to the protein surface and away from the substrate, increasing the available space in the substrate access channel and possibly allowing for a small substrate to enter (Figure 25) [41]. The orientation of Arg47 allows for the hydrophobic side chain of the decoy molecule to occupy this space and results in the water network being disturbed, increasing the hydrophobicity of the substrate access channel (Figure 25) [41]. Phe87 is a critical amino acid residue in CYP102 and is located in close proximity to the heme iron. Phe87 is perpendicularly oriented to the heme iron to reduce the steric conflict between the DMSO molecule (Figure 25) [41]. The perpendicular orientation of Phe87 can accommodate small alkanes near the heme iron. Collectively, these results provide crucial evidence that short-chain decoy molecules can “trick” CYP102, initiate the P450 catalytic cycle, and accommodate small alkanes within the active site.

#### 2.6.2. Dual-Functional Small Molecules (DFSMs)

Modifying the scaffolding of a molecule via selective C-H functionalization is implemented in the development of new chemicals, including pharmaceuticals. However, generating products from a substrate that possesses multiple sp3 and sp2 reaction sites remains an ongoing issue [42]. CYP102 can act as a peroxidase enzyme and therefore two dual-functional small molecules (DFSMs), namely, (N-imidazolyl)-hexanoyl-L-phenylalanine (IC6P) and (N-imidazolyl)pentanoyl-L-phenylalanine (IC5P), were designed to effectively activate the peroxide shunt pathway of CYP102 by hydrogen peroxide activation and utilizing substrates that affect the regio- and enantioselectivity of the enzyme [42]. Multiple amino acid residues were mutated and a number of variants were created to investigate this synthetic route.

**Figure 26:**
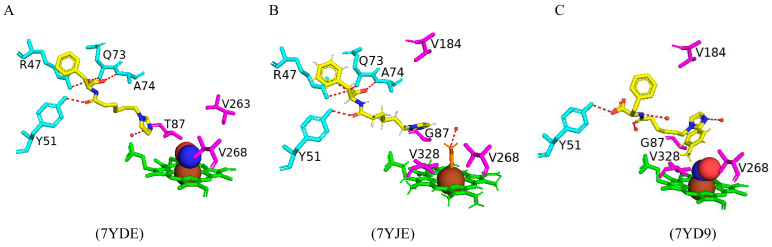
Active site cavity analysis of CYP102 mutants bound with one dual-functional small molecule (DFSM) and toluene. (**A**) CYP102 bound with (N-imidazolyl)-hexanoyl-L-phenylalanine (IC6P) and hydroxylamine. (**B**) CYP102 bound with IC6P and acetate. (**C**) CYP102 is bound with IC6P, hydroxylamine, and toluene. IC6P molecule is colored with carbon atoms as yellow, nitrogen as blue, and oxygen as red; heme is shown in green; the iron molecule is shown in brown; hydroxylamine is shown as blue and red spheres; acetate is shown in orange; toluene is shown in yellow. Mutated amino acid residues are shown in magenta, and amino acid residues sharing a polar interaction are shown in cyan. Polar interactions are indicated as red dashed lines; water molecules are shown as red dots, and amino acid residues are labeled according to their single-letter codes. PDB code is shown underneath the respective model. Amino acid residues within 5 Å of the DFSM and substrate are shown in Table S2.

Hydroxylamine or acetate was used instead of hydrogen peroxide to interact directly with the heme iron. Two variants, with multiple mutations, were crystallized with IC6P, and the DFSM bound with the terminal imidazolyl group oriented towards the heme iron (Figure 26A,B). Variant one has three mutations (F87T, T268V, I263V), and variant two has four mutations (F87G, A184V, T268V, A328V), and the latter variant was also bound with toluene (Figure 26). The crystal structure of variant one revealed direct hydrogen bonds between IC6P and four amino acid residues, namely, Arg47, Trp51, Gln73, and Ala74, and the imidazolyl group shares a direct hydrogen bond with a water molecule (Figure 26A). A similar hydrogen bonding pattern was observed in the second variant. However, the imidazolyl group does not interact with a water molecule. Instead, the acetate molecule interacts with a water molecule (Figure 26B). This may be due to the different orientation(s) of the imidazolyl group, possibly due to steric conflicts with the acetate molecule (Figure 26B) [42]. In the toluene-bound variant, the DFSM is in a “squashed” conformation, and only the hydrogen bond with Tyr51 remained, with three hydrogen bonds formed with three water molecules instead (Figure 26C). The A328V mutation maintained the proper conformation of DFSM within the variant enzyme [42]. The toluene molecule was bound in close proximity to the heme iron due to the F87G and A328V mutations, the imidazolyl group occupies this space in the absence of toluene, and therefore the imidazole ring can adopt multiple conformations during the catalytic cycle (Figure 26C) [42].

**Figure 27:**
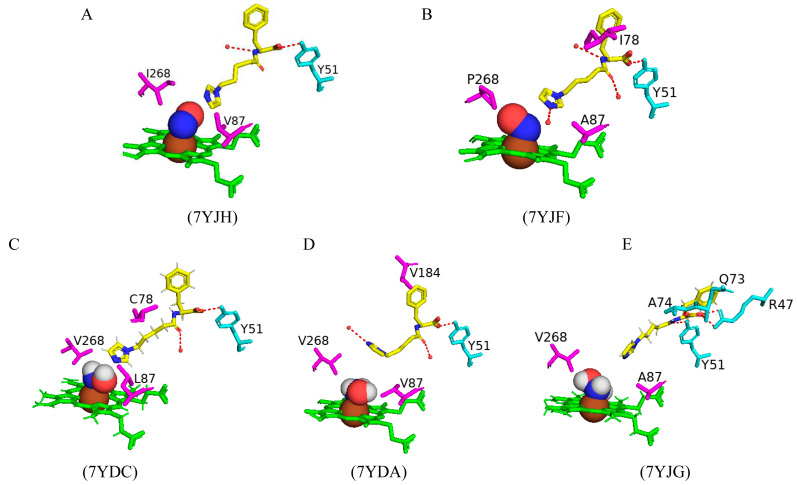
Active site cavity analysis of five CYP102 mutants bound with (N-imidazolyl)pentanoyl-L-phenylalanine (IC5P) and hydroxylamine. (**A**) Variant 3 bound with IC5P, (**B**) Variant 4 bound with IC5P, (**C**) Variant 5 bound with IC5P and hydroxylamine, (**D**) Variant 6 bound with IC5P and hydroxylamine, (**E**) Variant 7 bound with IC5P and hydroxylamine. IC5P molecule is colored with carbon atoms as yellow, nitrogen as blue, and oxygen as red; heme is shown in green; the iron molecule is shown in brown; hydroxylamine is shown as blue, red, and white spheres. Mutated amino acid residues are shown in magenta, and amino acid residues sharing a polar interaction are shown in cyan. Polar interactions are indicated as red dashed lines; water molecules are shown as red dots, and amino acid residues are labeled according to their single-letter codes. PDB code is shown underneath the respective model. Amino acid residues within 5 Å of the DFSM is shown in Table S2.

Five variants with multiple mutations were crystallized with IC5P and hydroxylamine. The majority of these mutations included amino acid residues Phe87 and Thr268 variants. Variant 3 has two mutations (F87V and T268V), variant 4 has three mutations (V78I, F87A, and T268P), variant 5 has three mutations (V78C, F87L, and T268V), variant 6 has three mutations (F87V, A184V, and T268V), and variant 7 has two mutations (F87A and T268V) (Figure 27). All variants shared a hydrogen bond with Tyr51, and the DFSM was bound in a similar orientation with the terminal imidazolyl group facing towards the heme iron (Figure 27). Variants one and two are similar structures. However, the mutation at Thr268 determines the orientation of the imidazole ring and may influence the interactions [42]. The imidazole ring in variant 3 does not share any polar interactions with the P450 apoprotein (Figure 27A). However, in the Pro268 mutation the imidazole ring orientation is tilted and shares a direct hydrogen bond with a water molecule (Figure 27B). Interestingly, in the Val268 mutation, the imidazole ring is almost parallel to the heme iron and retains a direct hydrogen bond with a water molecule (Figure 27C,D). Variant 7 shared a similar hydrogen bonding pattern with the same amino acid residues seen in variant 2 (Figure 27E). This highlights the flexibility of the imidazole ring and the different interactions associated with certain orientations.

**Figure 28:**
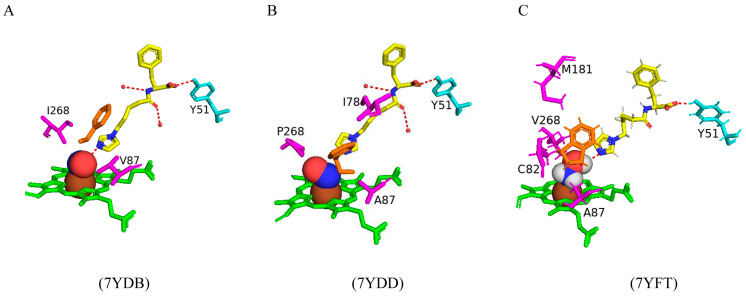
Active site cavity analysis of three CYP102 mutants bound with (N-imidazolyl)pentanoyl-L-phenylalanine (IC5P), hydroxylamine and ethylbenzene (**A**), propylbenzene (**B**), and indane (**C**). IC5P molecule is colored with carbon atoms as yellow, nitrogen as blue, and oxygen as red; heme is shown in green; iron molecule is shown in brown; hydroxylamine is shown in blue, red, and white spheres and substrates are shown in orange. Mutated amino acid residues are shown in magenta, and amino acid residues sharing a polar interaction are shown in cyan. Polar interactions are indicated as red dashed lines; water molecules are shown as red dots, and amino acid residues are labeled according to their single-letter codes. PDB code is shown underneath the respective model. Amino acid residues within 5 Å of the DFSM and substrate are shown in Table S2.

Variants 9–11 were crystallized with IC5P and ethylbenzene, propylbenzene, and indane, respectively. Variant 9 had two mutations (F87V and T268I), variant 10 had three mutations (V78I, F87A, and T268P), and variant 11 had four mutations (F87A, A82C, L181M, and T268V) (Figure 28A–C). IC5P shared the hydrogen bond with Tyr51 in all variants regardless of the cocrystallized substrate and was similarly oriented within the active site with the imidazole ring facing the heme iron (Figure 28A–C). The imidazole ring interacts directly with the hydroxylamine in both variants 9 and 11 (Figure 28A,C). This direct polar interaction is not seen in variant 10 as the propylbenzene molecule is located in very close proximity to the heme iron due to the Ala87 and Pro268 mutations as they provide more space and less steric conflicts compared to Val87, Ile268, and Val268, possibly perturbing this interaction (Figure 28B) [42]. The Cys82 and Met181 mutations in variant 11 led to the “squashed” conformation of IC5P (Figure 28C). The mutations in this variant were concentrated on one side of the heme iron, and this led to indane binding in a different conformation compared to variants 9 and 10 (Figure 28A,B). The orientation of ethylbenzene in variant 9 shows the pro-S hydrogen atom on the α-carbon to be closest to the heme iron and results in the formation of (S)-1-phenylethan-1-ol (Figure 28A) [42], whereas the orientation of the propylbenzene in variant 10 revealed the pro-S hydrogen atom on the β-carbon to be the closest to the heme iron and results in the formation of (S)-1-phenylpropan-2-ol (Figure 28B) [42]. Analysis of the indane-bound crystal structure revealed the pro-R hydrogen atom on the α-carbon to be closest to the heme iron and resulting in the formation of (R)-1-indanol (Figure 28C) [42]. The combination of CYP102 variants and DFSMs provided evidence of the cooperative effects during alkylbenzene hydroxylation and revealed controlled regio- and enantioselectivity at various sites [42]. These results highlight the power of CYP102 as an engineered P450 and the potential of DFSMs in facilitating enzymatic catalysis for biochemical transformations.

### 2.7. Cytochrome P411 Represents a Biocatalyst Able to Solve Challenging Selectivity Problems

**Figure 29:**
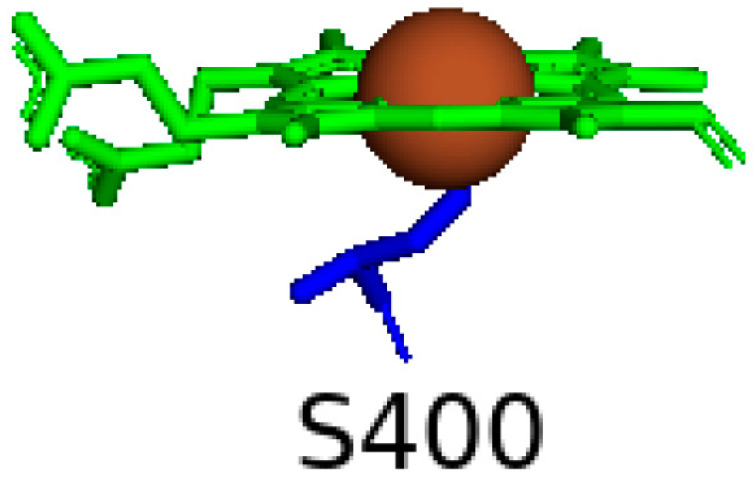
Active site cavity of a CYP102 mutant, cytochrome P411. Serine residue is shown in blue; heme is shown in green; iron molecule is shown in brown. The amino acid residue is labeled according to its single-letter code.

The ability to perform non-natural catalytic activities within cells will avail new biosynthetic pathways for both natural and synthetic products, as well as increase the scope of chemicals that may be used to investigate cellular function [37]. Cytochrome P411 enzymes were designed to catalyze efficient and selective olefin cyclopropanation in cells [37]. These enzymes had a serine residue in place of the conserved cysteine thiolate residue associated with CYP102 (Figure 29), with the aim to enhance the reduction potential of the heme protein. This helps facilitate NADPH-driven reduction and leads to an enhancement of catalysis [37]. Interestingly, P411 was able to catalyze efficient and selective carbene transfers from diazoesters to olefins [37]. The serine substitution led to a rise in the reduction potential and significantly improved NADPH-driven cyclopropanation activity [37]. Similarly, C–H amination activity is sensitive to the nature of the residue ligating the axial position of the heme iron [82]. The P411 variant of CYP102 revealed superior reactivity in the amination of secondary benzylic C–H bonds and has also been shown to be a proficient catalyst for nitrene transfer. P411 also can aminate benzylic C–H bonds in diverse structures with high selectivity [83]. Ultimately these enzymes can serve as starting points from which to evolve C–H alkylases to divergently functionalize C(sp3)–H and C(sp2)–H bonds with high selectivity [84].

### 2.8. CYP102 Serves as a Model Enzyme to Study Structural Dynamics Using a Cost-Effective Crystallization Accelerator

The abietane diterpenoid derivative N-abietoyl-l-tryptophan (AbiATrp) has been shown to have great influence on the speed of crystallization of the CYP102 heme domain with visible crystals forming within 2 h [43]. In contrast, a dehydroabietic acid modified with tryptophan, known as a common analog of AbiATrp, did not prompt crystallization to the same extent as AbiATrp [43]. This may be explained by the low solubility of AbiATrp in aqueous solution, indicating that AbiATrp remains bound within the hydrophobic channel of CYP102 and does not dissociate and rebind [43].

**Figure 30:**
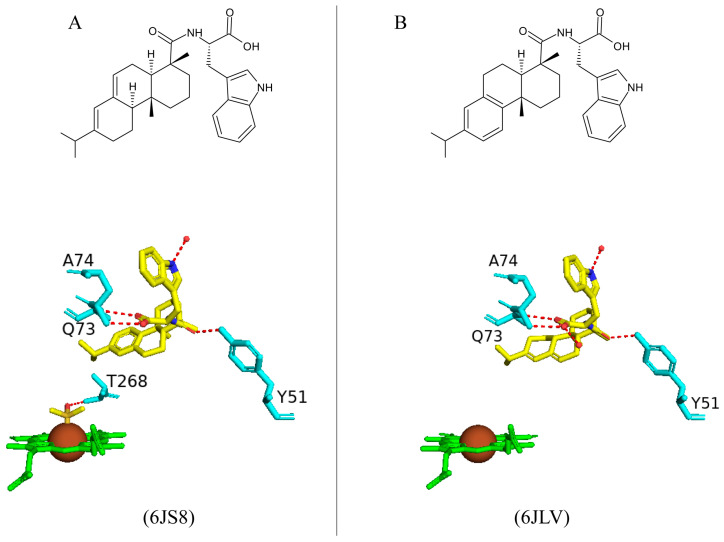
Structural analysis of CYP102 bound with a crystallization accelerator and its structural analog. Abietane diterpenoid derivative N-abietoyl-l-tryptophan (**A**) and dehydroabietic acid modified with tryptophan (**B**). Ligand is colored with carbon atoms as yellow, nitrogen as blue, and oxygen as red; heme is shown in green; the iron molecule is shown in brown, and the solvent molecule is colored with carbon atoms as yellow, sulfur as light brown, and oxygen as red. Amino acids sharing a polar interaction are shown in cyan. Polar interactions are indicated as red dashed lines; water molecules are represented as red dots, and amino acid residues are labeled according to their single-letter codes. PDB code is shown underneath the respective model. The chemical structures of the ligands are shown above the protein models, and the PDB code is shown underneath the respective model. Amino acid residues within 5 Å of the ligand are shown in Table S2.

AbiATrp binds within the CYP102 active site in a curved orientation with a methyl group pointing towards the heme iron (Figure 30A). A DMSO molecule is bound to the heme iron and shares a direct hydrogen bond with Thr268 (Figure 30A). The nitrogen atom on the pentane ring shares a direct hydrogen bond with a water molecule, and the hydroxyl group shares a direct hydrogen bond with Tyr51, whereas the oxygen atom shares two direct hydrogen bonds with Gln73 and Ala74 (Figure 30A). These polar interactions are identical to the interactions seen between N-palmitoylglycine and CYP102 (Figure 3B). The orientation of the structural analogue is similar to AbiATrp and shares the same polar interactions as AbiATrp, with the only difference being the absence of the DMSO molecule (Figure 30B).

**Figure 31:**
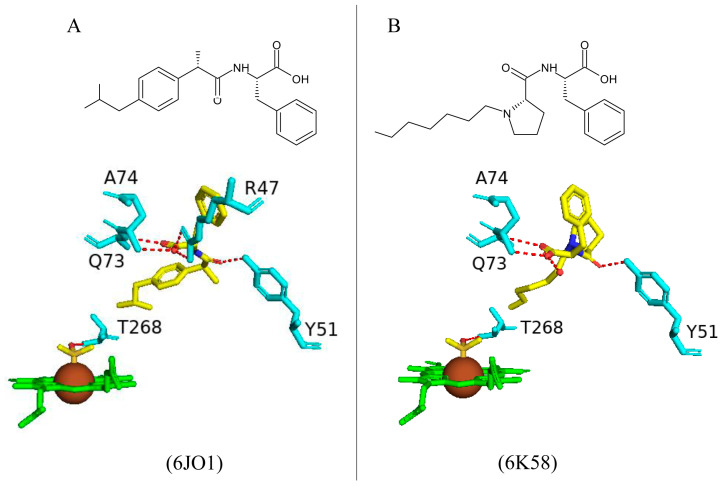
CYP102 bound with two decoy molecules crystallized with CYP102–AbiATrp seeds. N-(S)-ibuprofenoyl-l-phenylalanine (**A**) and N-enanthyl-l-prolyl-l-phenylalanine (**B**). Ligand is colored with carbon atoms as yellow, nitrogen as blue, and oxygen as red, heme is shown in green, the iron molecule is shown in brown, and the solvent molecule is colored with carbon atoms as yellow, sulfur as light brown, and oxygen as red. Amino acids sharing a polar interaction are shown in cyan. Polar interactions are indicated as red dashed lines; water molecules are represented as red dots, and amino acid residues are labeled according to their single-letter codes. PDB code is shown underneath the respective model. The chemical structure of the ligand is shown above the protein model, and the PDB code is shown underneath the respective model. Amino acid residues within 5 Å of the ligand is shown in Table S2.

Crystallization of the CYP102 heme domain bound with decoy molecules has been challenging with only a few successful crystal structures determined. However, crystals of CYP102 in complex with two decoy molecules, N-(S)-ibuprofenoyl-l-phenylalanine (SIbuPhe) and N-enanthyl-l-prolyl-l-phenylalanine (C7ProPhe), formed within seconds upon the addition of CYP102-AbiATrp seeds [43]. These two decoy molecules are well-suited to the hydroxylation of benzene [43]. SIbuPhe is bound within the active site of CYP102 in a similar orientation to AbiATrp, with the methyl group pointing towards the heme iron (Figure 31A). SIbuPhe shares direct hydrogen bonds with the hydroxyl group of Tyr51 and the amide moieties of Gln73 and Ala74 whereas Arg47 shared a bidentate hydrogen bond with the carboxylate group of SIbuPhe (Figure 31A). As seen in the AbiATrp-bound structure, a DMSO molecule was bound to the heme iron and shared a direct hydrogen bond with Thr28 (Figure 31A). C7ProPhe bound within the CYP102 active site in a similar orientation to SIbuPhe, with a DMSO molecule bound to the heme iron and sharing identical polar interactions excluding Arg47 (Figure 31B). It is interesting to note that C7ProPhe exhibited a high catalytic activity for the hydroxylation of benzene and this can be attributed to the basic tertiary amine group forming an intramolecular hydrogen bond with the hydrogen atom of its own proline–phenylalanine peptide amide group rather than with CYP102 directly [43]. This interaction may control the motion of C7ProPhe and ensure that it is oriented in a favorable position to bind to CYP102. These results offer excellent routes for the further design of potent specific decoy molecules for CYP102 and for other P450s in general.

**Figure 32:**
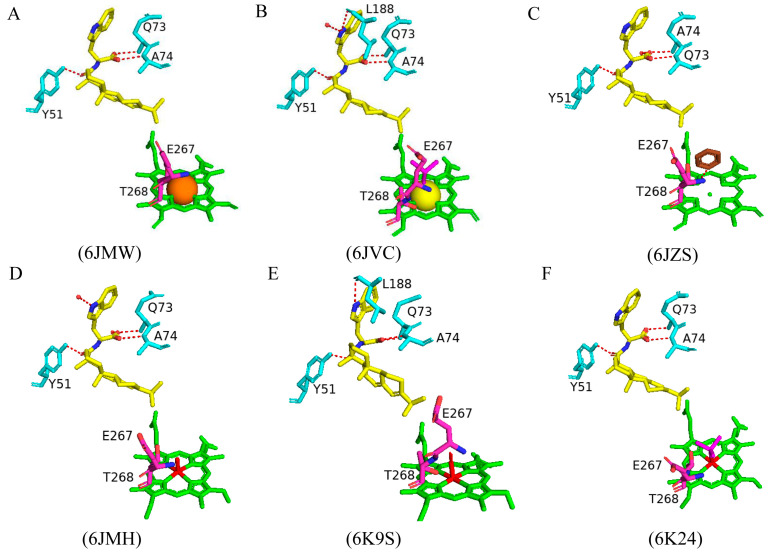
CYP102 bound with AbiATrp and six metalloporphyins. Chromium protoporphyrin IX (CrPPIX) (**A**), cobalt protoporphyrin IX (CoPPIX) (**B**), manganese protoporphyrin IX (Mn(py)PPIX) (**C**), oxymolybdenum mesoporphyrin IX (Mo(O)MPIX) (**D**), carbonylruthenium mesoporphyrin IX (Ru(CO)MPIX) (**E**), and rhodium mesoporphyrin IX (RhMPIX) (**F**). AbiATrp is colored with carbon atoms as yellow, nitrogen as blue, and oxygen as red; heme is shown in green; Chromium is shown as an orange sphere, cobalt as a yellow sphere, manganese in brown, 4d transition metals are shown as red sticks, and solvent molecules are shown as magenta. Glu267 and Thr268 are colored with carbon atoms as pink, nitrogen as blue, and oxygen as orange. Amino acids sharing a polar interaction are shown in cyan. Polar interactions are indicated as red dashed lines; water molecules are represented as red dots, and amino acid residues are labeled according to their single-letter codes. The PDB codes are shown underneath the respective models. Amino acid residues within 5 Å of AbiATrp are shown in Table S2.

There is a lack of resolved CYP102 crystal structures cocrystallized with artificial cofactors. However, using CYP102–AbiATrp mixture seeds, six crystal structures of CYP102 containing artificial cofactors were produced with transition metals including chromium, manganese, cobalt, molybdenum, ruthenium, and rhodium [43]. In the crystal structure of CYP102 and chromium protoporphyrin IX (CrPPIX), AbiATrp oriented in a similar fashion with the methyl group pointing towards the CrPPIX and sharing the same polar interactions with Trp51, Gln73, and Ala74 (Figure 32A). Interestingly, a DMSO molecule was bound to the cobalt protoporphyrin IX (CoPPIX) and the nitrogen atom shares a hydrogen bond with a water molecule and Leu188 (Figure 32B). The crystal structure of CYP102 and manganese protoporphyrin IX (Mn(py)PPIX) showed the same polar interactions and orientation as CrPPIX but revealed a fascinating observation as the manganese atom was displaced out of the porphyrin plane, a characteristic associated with a high-spin iron atom (Figure 32C) [43]. This structure may illustrate the first step in the CYP102 catalytic cycle.

Analysis of the crystal structures of CYP102 bound with 4d transition metals, oxymolybdenum mesoporphyrin IX (Mo(O)MPIX), carbonylruthenium mesoporphyrin IX (Ru(CO)MPIX), and rhodium mesoporphyrin IX (RhMPIX) revealed similar orientations of the crystallization accelerator with similar polar interactions with amino acid residues Trp51, Gln73, and Ala74 (Figure 32D–F). The slight difference noticed was the nitrogen atom sharing a hydrogen bond with a water molecule in the (Mo(O)MPIX)-bound structure, which was also observed in CoPPIX (Figure 32D). It has been suggested that CYP102 bound with Mo(O)MPIX represents an excellent model for the heme environment of CYP102–Cpd II [43]. The same nitrogen atom shares a direct hydrogen bond with Leu188 instead of a water molecule in the (Ru(CO)MPIX) structure, which is also observed in the CoPPIX structure (Figure 32E). Finally, analysis of the (RhMPIX) structure revealed a DMSO molecule bound to the rhodium moiety; and this was also observed in the CoPPIX structure (Figure 32F) [43].

A closer look at the structural architecture of CYP102 bound with each metalloporphyin revealed different conformations of Thr268, which is known to be involved in proton delivery, dioxygen activation, and catalytic intermediate stabilization [43]. In the Mn(py)PPIX structure, both Thr268 and Glu267 are bound in an “up” conformation which is associated with the hydroxyl group of Thr268 pointing away from the protoporphyrin IX (Figure 32B). In the Ru(CO)MPIX structure, the opposite was observed, with both Glu267 and Thr268 adopting a “down” conformation with the hydroxyl group of Thr268 pointing downwards towards the protoporphyrin IX (Figure 32E). Some structures showed a combination of both conformations for these two amino acid residues, such as observed in Mo(O)MPIX and CoPPIX [43]. The use of this crystallization accelerator has yielded high-resolution crystal structures that have revealed critical amino acid interactions, as well as the possibility of using these model enzymes to probe the structural dynamics of the CYP102 protein during the catalytic cycle [43].

### 2.9. Cost-Effective Techniques to Eradicate the Use of NADPH as a Potential Strategy for Industrializing CYP102

#### 2.9.1. Ru(II)-Diimine Photosensitizers

**Figure 33:**
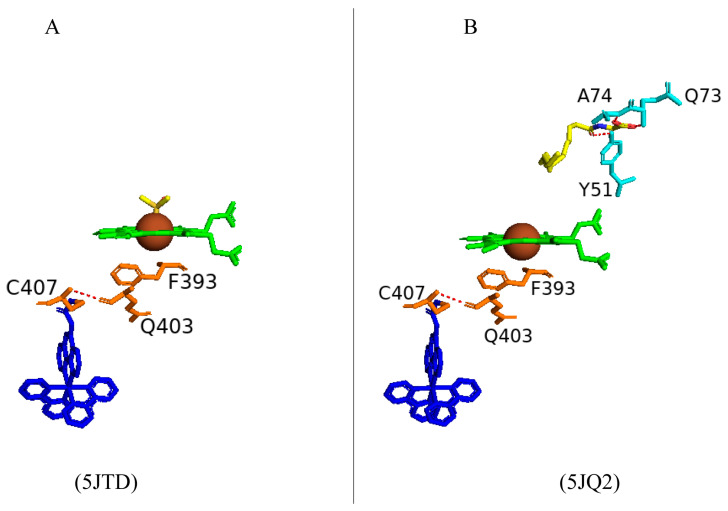
Structural analysis of sL407C-1, a Ru(II)-diimine functionalized hybrid of CYP102. sL407C-1 in open conformation (**A**) and sL407C-1 in a closed conformation (**B**). Photosensitizer is shown in blue; ligand is colored with carbon atoms as yellow, nitrogen as blue, and oxygen as red; solvent molecule is shown in magenta; heme is shown in green; iron is shown as a brown sphere. Amino acids sharing a polar interaction with the photosensitizer are shown in orange and amino acids sharing a polar interaction with the ligand are shown in cyan. Polar interactions are indicated as red dashed lines, and amino acid residues are labeled according to their single-letter codes. The PDB code is shown underneath the respective model. Amino acid residues within 5 Å of the ligand are shown in Table S2.

To reduce the generation of reactive oxygen species due to uncoupled pathways and to ensure high catalytic efficiency during the P450 catalytic cycle, the successive delivery of electrons must be carefully coordinated [68]. To avoid using reductase and a cofactor, many studies have focused on developing alternative approaches to drive the P450 enzyme and deliver electrons successfully. P450 fusion proteins and light-activated approaches are examples of such approaches [85]. Ru(II)-diimine photosensitizers are well-known for their photochemical properties and their substantial electron transfer abilities in metalloproteins [85]. sL407C-1, a Ru(II)-diimine functionalized hybrid of CYP102, has been developed to perform selective C-H functionalization via visible light irradiation as a source of reducing equivalents [85]. The crystal structure of the open conformation of the sL407C-1 enzyme reveals the covalent attachment of the photosensitizer to an introduced non-native cysteine (L407C), and it is in the same position as the FMN cofactor in wild-type CYP102 (Figure 33A). The photosensitizer shared π interactions with several protein residues, which probably plays a role in stabilizing the P450 tertiary structure and molecular recognition [85]. sL407C-1 Cys407 shares a direct hydrogen bond with Gln403, and a DMSO molecule was bound to the heme iron via its sulfur atom (Figure 33A). N-palmitoylglycine was crystallized to the hybrid enzyme, positioned within the active site of CYP102, in a similar manner to the wild-type enzyme, and shared the same polar interactions with amino acid residues such as Tyr51, Gln73, and Ala74 (Figure 3B and Figure 33B). The photosensitizer shared the same polar interactions in both open and closed conformations and did not affect the overall tertiary fold of CYP102 (Figure 33).

The covalent attachment to the non-native single cysteine (L407C) of the CYP102 hybrid enzyme permits intramolecular electron transfer (Figure 33) [85]. The photosensitizer in the sL407C-1 enzyme is positioned in a way that makes it possible for it to rapidly transfer electrons to the heme iron via the naturally occurring electron transfer pathway including the highly conserved Phe393 and Gln403 residues, activating molecular dioxygen and maintaining photocatalytic activity [85]. Mutations of these two residues in the sL407C-1 hybrid enzyme resulted in the generation of three mutants, namely, F393A, F393W, and Q403W. The Q403W mutant was designed to increase the electronic communication between the photosensitizer and the heme through the staggering of multiple aromatic rings [85].

Spectroscopic absorption analysis revealed rapid electron transfer from the photosensitizer to the heme moiety upon visible light irradiation because the heme iron was reduced in all mutant enzymes within 100 microseconds after the laser pulse [85]. Despite the quick delivery of electrons to the heme iron, colorimetric assay studies revealed negligible photocatalytic activity for the F393A mutant and a 20% decrease in activity in the F393W mutant enzyme compared to the sL407C-1 hybrid enzyme [85]. The low/lack of photocatalytic activity indicates a high degree of uncoupling during electron delivery. Interestingly, the Q403W mutant revealed a 60% increase in photocatalytic activity due to improved electronic coupling between the photosensitizer and the heme domain [85]. These results indicate that the hybrid enzyme’s photosensitizer is well-positioned and can successfully and rapidly deliver electrons to the heme iron using the electron transfer pathway that naturally occurs in CYP102. Mutations of the amino acid residues within the naturally occurring electron transfer pathway indicated the important role they play in the enzyme’s photocatalytic activity. These results represent new avenues for the future development of light-driven P450 biocatalysts.

#### 2.9.2. Zinc/Cobalt (III) Sepulchrate (Co(III)Sep) as an Electron Source to Drive CYP102 Activity

**Figure 34:**
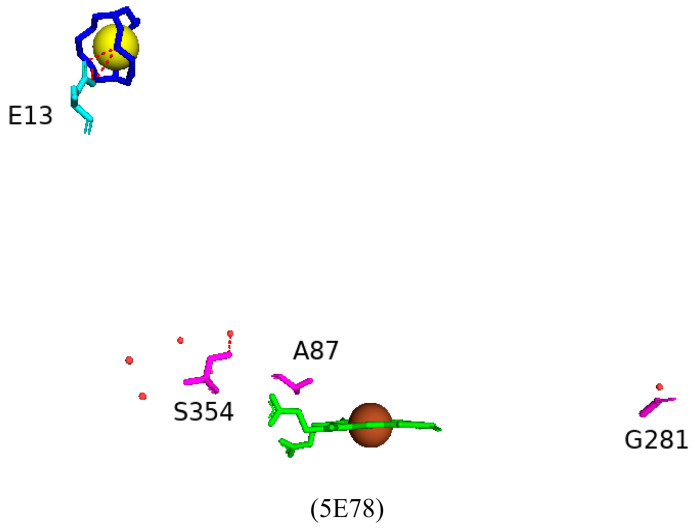
CYP102 heme domain bound with cobalt (III) sepulchrate. Heme is shown in green; iron is shown as a brown sphere; cobalt (III) sepulchrate is shown in blue with the cobalt atom shown as a yellow sphere. Amino acids sharing a polar interaction with cobalt (III) sepulchrate are shown in cyan and substituted amino acids are shown in magenta. Polar interactions are indicated as red dashed lines; water molecules are shown as red dots, and amino acid residues are labeled according to their single-letter codes. The PDB code is shown underneath the respective model. Amino acid residues within 5 Å of the mediator are shown in Table S2.

The need for alternative cofactors stems from the NADPH requirement of CYP102, which limits the application of CYP102 in drug detection and synthesis, primarily due to cost. Another example of an alternative cost-effective cofactor system is the zinc/cobalt (III) sepulchrate (Co(III)Sep) system [86]. Zinc dust is known as an electron source for CYP102 with Co(III)Sep serving as a mediator as the electronic communicator [86]. Many variants of CYP102 have been designed to probe the role of various residues and increase favorable interactions between CYP102 and a cofactor. The M7 variant has shown an increased activity compared to the wild-type enzyme (76% increase in activity) [86]. The M7 variant consists of three substitutions, namely, F87A, V281G, and M453S, and has shown a preference for the Zn/Co(III)Sep cofactor (Figure 34). The crystal structure of CYP102 and cobalt (III) sepulchrate reveals an unusual binding site for the mediator, located towards the mouth of the substrate access channel (Figure 34). The mediator is positioned far away from the active site and heme iron and is uniquely different when compared to the binding site of the natural redox partner (FAD/NADPH-binding domain). The mediator binds at the distal face of the heme domain which is electrostatically favorable as the overall distal surface is negatively charged and the Co(III)Sep is positively charged [86]. The FMN domain is positioned at the proximal surface of the heme domain which is positively charged, therefore, due to unfavorable electrostatic interactions between the proximal surface and the mediator, the mediator was bound at the negatively charged distal surface [86]. Unlike the sL407C-1 hybrid enzyme, the M7 variant is not covalently attached to the mediator indicating a probable novel binding site [86]. However, due to the large distance between the Co(III)Sep and heme iron in the crystal structure, direct interactions or electron transfers cannot occur and, thus, the mediator’s binding may be attributable to the crystallization preparation procedure.

Substitutions of three amino acid residues within the active site of CYP102 revealed their possible mechanistic roles, promoting catalytic activity using the Zn/Co(III)Sep cofactor system. F87A enlarges the space volume in the active site and encourages substrate entry into the heme center. This amino acid residue is known to be involved in substrate recognition and orientation. V281G potentially improves crystal packing of this structure, as a water molecule replaces the space generated by this substitution (Figure 34) [86]. Despite this, this mutation did not lead to any significant changes to the overall structure of CYP102. Water molecules surround the serine residue in the M453S substitution and share a direct hydrogen bond with one water molecule (Figure 34). It is hypothesized that upon substrate binding these water molecules are displaced, and Ser354 forms favorable interactions that lead to the stabilization of the substrate within the active site [86]. These results highlight the structural selectivity of the CYP102 cofactor and indicate that the positive charge of Co(III)Sep is vital for its efficient binding to the heme domain.

## 3. Materials and Methods

### 3.1. Retrieving of CYP102 Members’ Structures

A hundred and thirty-six CYP102 protein crystal structures were retrieved from the Research Collaboratory for Structural Bioinformatics Protein Data Bank (RCSB PDB) [44]. Only 105 crystal structures are available for public use, and their structures were published and used in the study (Table S3). It is important to note that all structures of CYP102 available in the RCSB PDB belong to subfamily A.

### 3.2. CYP102 Active Site Analysis

CYP102 active site analysis was performed following methodology recently developed and described by our laboratory [46]. Briefly, individual CYP102 crystal structures were analyzed and assigned to either an open conformation (containing only heme cofactor) or a closed conformation (bound with ligand and heme). Each CYP102 crystal structure’s area and volume were analyzed for both open and closed conformations using the Computed Atlas of Surface Topography of Proteins (CASTp) database version 3.0 [87]. In this study, we used the CASTp program for active site cavity analysis because the other articles describing P450 structure–activity analysis, such as that of CYP107 [45] and CYP121 [46], also used the same program. Using the same program facilitates accurate comparison of active site cavity volumes among different P450s. For active site analysis, each PDB file was individually uploaded onto PyMOL software, Version 2.2.5 [88]. The active site cavities were selected using heme as the center point of the binding pocket and amino acid residues within 5 Å were chosen. The amino acid residues were recorded for both open and closed conformations. Consequently, conserved and critical amino acids within the open and closed conformation were identified. The active site is represented by the heme in open conformation and the heme and other ligands in closed conformation within the binding pocket. The amino acid residues are represented as sticks and labeled using the one-letter amino acid codes.

### 3.3. Analysis of Ligand Interactions in Closed Conformation PDB Files Using PyMOL

Fifty-seven of the one hundred and five crystal structures studied were in the closed conformation. Firstly, individual PDB files were uploaded into PyMOL, and the active site cavity was selected, as mentioned in Section 3.2. If the bound ligand extended out of the selected binding pocket, 5 Å from the ligand was chosen to include all interacting amino acid residues. The amino acids are represented as sticks and labeled according to their one-letter amino acid code. Polar contacts with any atoms were then selected. If ligand interactions with specific amino acid residues were determined, dashed lines connected the ligand and the specific amino acid residue, water molecule, or solvent molecule. For data obtained from published literature, hydrophobic residues within 5 Å were also selected and shown as sticks. The amino acid residues that were not interacting with the ligand were removed.

### 3.4. Annotation of P450 Characteristic Secondary Structures and Substrate Recognition Sites (SRSs)

P450 characteristics and identities for CYP102 alpha helices and beta sheets were examined as described elsewhere [89]. The CYP102 PDB file (2HPD) was chosen and uploaded into PyMOL software, Version 2.2.5 [88]. The alpha helices and beta sheets were assigned different colors, which were used to map the secondary structural elements of the chosen protein sequence. Subsequently, alpha helices and beta sheets were named following published data [89]. The identification of CYP102 substrate recognition sites (SRSs) was carried out as described elsewhere [90]. Briefly, SRS1 was mapped between alpha helices B and C along the BC-loop, SRS2 is located in the C-terminal end of alpha helix F, SRS3 and SRS4 are located by the N-terminal regions of alpha helix G, SRS5 is found within beta sheets 1–4, and SRS6 is located in beta sheets 4–1. 

## 4. Conclusions

**Figure 35:**
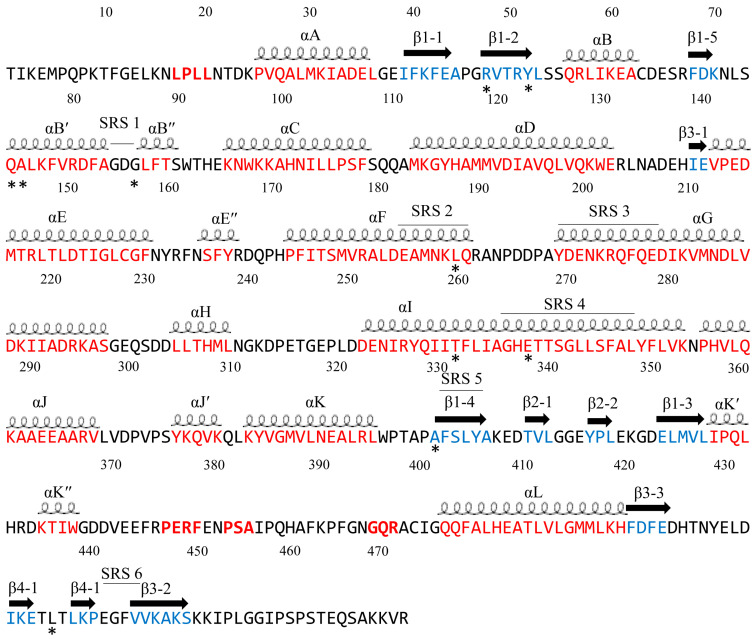
P450 characteristics and secondary structural analysis of CYP102A. Alpha helices are shown as coils, the amino acid residue part of alpha helices is highlighted in red, beta sheets are shown as black arrows, and beta sheets are highlighted in blue. Amino acid residues involved in direct polar interactions with ligands are represented by (*) underneath the respective residue. P450 characteristic naming for alpha helices, beta sheets, and identifying substrate recognition sites (SRS) was performed following the methods described elsewhere [89,90].

CYP102 displays high substrate specificity towards fatty acids whilst remaining highly flexible. Additionally, this P450 was found to have the greatest overall structural changes (highest RMSD value) compared to other reported P450s such as CYP121 and CYP107. A closer examination of the CYP102 active site revealed various amino acid residues located within close proximity to the heme iron that is directly involved in altering the substrate specificity of the enzyme and providing a clear path toward the reactive heme iron center. Unlike most P450s, substrate binding was not required for the conformational equilibrium shift observed in CYP102. Movement of the “lid” domain encompassing the F and G helices, the I helix, and the B’ helix causes a narrowing in the substrate access channel and thus shifts the equilibrium to a closed conformation. Changes in CYP102 structural conformation are not merely due to the displacement of the sixth water ligand but also the interaction with Ala264. Analysis of CYP102 bound to palmitoleic acid and N-palmitoylglycine revealed Tyr51 to act as a substrate anchor within the active site, and Arg47 is essential for the initial binding of N-palmitoylglycine during the first catalytic steps. Decoy molecules are a promising approach to evade the substrate specificity of CYP102 and permit small non-native substrates access to the heme iron. Removing the need for NADPH, an expensive cofactor, is a potential strategy to industrialize CYP102. A crystallization accelerator yielded high-resolution crystal structures of CYP102 that revealed many critical interactions within the active site. Interestingly, mapping of the CYP102 sequence revealed interactions between ligands and the protein found in four of the six substrate recognition sites (Figure 35).

Mutations of amino acid residues of CYP102 revealed critical information regarding active site dynamics, substrate specificity, and reactivity. Thr268 is conserved in all CYP102s known to date, and mutation of this residue results in an enzyme that is catalytically inefficient. Phe87 is involved in substrate specificity and protects the heme from the solvent. Certain mutations cause steric hindrances that affect the enantioselectivity of CYP102. Mutations within the substrate access channel decreased the energetic barrier and permitted the conformational change from open to closed forms. Conformational destabilization enables CYP102 to explore novel conformations and accept diverse substrates. On the other hand, variants of CYP102 led to a destabilized secondary structure, which may be solved by mutations that increase the hydrophobic interactions and salt bridge networks within the protein. Site-directed mutagenesis significantly improved CYP102’s performance as a ligand-responsive MRI sensor. CYP102 variant enzymes have strong potential to be industrial biocatalysts and serve as unique templates for future P450 protein-engineering approaches that will selectively metabolize drugs.

This study provides comprehensive information on CYP102 structure–function analysis and reveals new insights regarding active site cavity dynamics and specific amino acids’ roles in catalysis. This work will contribute to the further improvement and engineering of CYP102 to accommodate and catalyze non-native substrates to be ultimately industrialized.

## Figures and Tables

**Table 1:** Comparative analysis of the surface area, volume, and RMSD of three P450s.

P450 Name	Change in Average Surface Area (Å^2^)	Change in Average Volume (Å^3^)	RMSD (Å)	Reference
CYP107FH5	276	494	3.0	[45]
CYP121A1	37	8	0.225	[46]
CYP102A1	179	23	4.4	Current work

**Table 2:** Analysis of amino acid dynamics for CYP102. Amino acid residues within 5 Å of the heme were selected. Amino acids that are conserved, unique, and part of the active site are presented.

Conformation	Number of Crystal Structures	Amino Acids Part of the Active Site (Number of Amino Acids)	Conserved Amino Acid Residues (Number of Amino Acids)	Unique Amino Acids (Number of Amino Acids)
Open	46 *	Lys69, Asn70, Leu75, Leu86, Phe87, Trp96, His100, Phe107, Ile153, Leu233, Thr260, Phe261, Ala264, Gly265, His266, Thr268, Thr269, Leu272, Leu322, Pro326, Thr327, Ala328, Pro329, Ala330, Phe331, Ser332, Ile357, Lys391, Pro392, Phe393, Gly394, Asn395, Gln397, Arg398, Ala399, Cys400, Ile401, Gly402, Gln403, Phe405, Ala406, Leu407 (42)	Lys69, Asn70, Leu75, Leu86, Phe87, Trp96, His100, Phe107, Ile153, Thr260, Phe261, Ala264, Gly265, Thr268, Thr269, Leu272, Leu322, Thr327, Ala328, Phe331, Ser332, Ile357, Pro392, Phe393, Gly394, Gln397, Arg398, Ala399, Cys400, Ile401, Gly402, Phe405, Ala406 (33)	None
Closed	47 **	Lys69, Asn70, Trp72, Leu75, Leu86, Phe87, Trp96, His100, Phe107, Ile153, Leu233, Gln257, Thr260, Phe261, Ala264, Gly265, His266, Thr267, Thr268, Thr269, Leu272, Leu322, Pro326, Thr327, Ala328, Ala330, Phe331, Ser332, Ile357, Lys391, Pro392, Phe393, Gly394, Asn395, Gln397, Arg398, Ala399, Cys400, Ile401, Gly402, Gln403, Phe405, Ala406, Leu407, Glu409, Leu437 (45)	Lys69, Leu75, Leu86, Phe87, Trp96, His100, Phe107, Ile153, Thr260, Phe261, Ala264, Gly265, Thr268, Thr269, Leu272, Leu322, Thr327, Ala328, Phe331, Ser332, Ile357, Pro392, Phe393, Gly394, Gln397, Arg398, Ala399, Cys400, Ile401, Gly402, Phe405, Ala406 (31)	ATrp72, Gln257, Thr267 (3)

* Two structures were not included in the analysis as no heme was present (4DQK and 4DQL). ** Ten structures were not included in the analysis as a crystallization accelerator was used (6JS8, 6K58, 6JZS, 6JMW, 6JVC, 6K24, 6JMH, 6JLV, 6K9S, 6JO1).

## Data Availability

Data are contained within the article.

## Supplementary Materials

The supporting information can be downloaded at: https://www.mdpi.com/article/10.3390/ijms26052161/s1.
